# Respiratory Dendritic Cell Subsets Differ in Their Capacity to Support the Induction of Virus-Specific Cytotoxic CD8^+^ T Cell Responses

**DOI:** 10.1371/journal.pone.0004204

**Published:** 2009-01-15

**Authors:** Taeg S. Kim, Thomas J. Braciale

**Affiliations:** 1 Beirne B. Carter Center for Immunology Research, University of Virginia, Charlottesville, Virginia, United States of America; 2 Departments of Microbiology and Pathology, University of Virginia, Charlottesville, Virginia, United States of America; New York University School of Medicine, United States of America

## Abstract

Dendritic cells located at the body surfaces, e.g. skin, respiratory and gastrointestinal tract, play an essential role in the induction of adaptive immune responses to pathogens and inert antigens present at these surfaces. In the respiratory tract, multiple subsets of dendritic cells (RDC) have been identified in both the normal and inflamed lungs. While the importance of RDC in antigen transport from the inflamed or infected respiratory tract to the lymph nodes draining this site is well recognized, the contribution of individual RDC subsets to this process and the precise role of migrant RDC within the lymph nodes in antigen presentation to T cells is not clear. In this report, we demonstrate that two distinct subsets of migrant RDC - exhibiting the CD103^+^ and CD11b^hi^ phenotype, respectively - are the primary DC presenting antigen to naïve CD4^+^ and CD8^+^ T lymphocytes in the draining nodes in response to respiratory influenza virus infection. Furthermore, the migrant CD103^+^ RDC subset preferentially drives efficient proliferation and differentiation of naive CD8^+^ T cells responding to infection into effector cells, and only the CD103^+^ RDC subset can present to naïve CD8^+^ T cells non-infectious viral vaccine introduced into the respiratory tract. These results identify CD103^+^ and CD11b^hi^ RDC as critical regulators of the adaptive immune response to respiratory tract infection and potential targets in the design of mucosal vaccines.

## Introduction

A critical initial event in the induction of adaptive immune response is the uptake and presentation of antigen by dendritic cells (DC) to adaptive immune cells. DC reside within organized secondary lymphoid organs (e.g., spleen, lymph nodes, Peyer's Patches) and also localize at body surfaces (e.g., the skin, gastrointestinal and respiratory tracts) where they sample the external environment for foreign antigens and invading infectious microorganisms [1], [2], [3], [4]. At body surfaces, DC normally display an immature phenotype. Inflammatory stimuli such as infection trigger a sequence of maturation events resulting in the migration of the activated antigen-bearing DC to secondary lymphoid organs, e.g., lymph nodes (LN), via lymphatic vessels draining the inflamed body surface along a chemokine-dependent gradient [5], [6]. It is within the draining lymph nodes (DLN) that adaptive immune responses are initiated [7], [8]. The DC populations present in DLN include not only migrant antigen-bearing DC and DC resident in the lymphoid organs [3], but also DC in the circulation, i.e., plasmacytoid DC (pDC) [9], [10], [11] and Gr-1^+^ monocytic DC (MoDC) derived from circulating Gr-1^hi^ monocyte precursors [12], [13], [14], which enter the DLN directly from the circulation or first enter the peripheral sites of infection in response to inflammatory stimuli. After differentiation/maturation, these inflammation-induced DC can also capture antigens at the sites of infection and migrate to the DLN.

DC found both within lymphoid organs and in peripheral tissues are composed of multiple cell subsets [2], [3]. The subsets are defined based largely on the differential expression of specific cell surface markers, but also on the anatomic distribution of particular DC population and on the role or function of the DC subset as innate immune effector cells or in the induction of adaptive immune responses. For example, CD8αα^+^ DC found in secondary lymphoid organs are believed to play a dominant role in cross-presentation of non-replicating antigens, e.g., soluble proteins, to naïve CD8^+^ T cells [15], [16]. In the normal (non-inflamed) respiratory tract, several distinct subsets of RDC have been defined [17], [18], [19], [20]. RDC form an interdigitating network along the epithelium and mucosa of large (conducting) and small airways. They also reside as well throughout the lung parenchyma and occasionally within airways. Recently, two subsets of the CD11c^hi^ MHC II^hi^ RDC with anatomically distinct distributions have been identified [19]: one of the RDC subsets is defined by expression of the αE integrin CD103 (CD103^+^ RDC) and preferentially localizes to airway mucosa (and pulmonary vessels); the second subset, CD103^−^CD11b^hi^ DC (CD11b^hi^ RDC), localizes to the submucosa and the lung parenchyma. Recent evidence suggests that different RDC subsets may be involved in the induction of T cell responses in the respiratory tract [19], [21], [22], but role of these two MHC II^hi^ RDC subsets as well as the contribution of the more prominent Gr-1^+^ monocytic DC (MoDC) in the host response to respiratory infection is largely unknown.

Experimental influenza virus infection of the respiratory tract triggers accelerated migration of RDC out of the respiratory tract into the DLN and induces a vigorous CD4^+^ and CD8^+^ T cell responses [7], [8], [23], [24]. The contribution of RDC in general and specific subsets of RDC in viral antigen presentation to and activation of naïve T cells is not well understood. Several lines of evidence, however, suggest that tissue DC, such as RDC, in response to infection or inflammation may primarily serve in antigen transport to the DLN where LN-resident DC acquire antigen from migrant tissue-derived DC and cross-present it to T cells [7], [25], [26]. In this report, we characterized the DC residing in the respiratory tract – before and after influenza infection – and assessed the ability of specific subsets of migrant RDC to activate influenza virus-specific CD8^+^ and CD4^+^ T cells in response to infection and non-infectious virus vaccine introduced into the respiratory tract.

## Results

### Respiratory dendritic cell subsets and virus infection

DCs are found both in lymphoid organs and in peripheral sites, e.g. skin, lungs, etc., and are composed of distinct subsets. We employed a 6 color flow-cytometric strategy to identify RDC subsets among the heterogeneous population of CD45^+^CD11c^+^ cells in the normal (non-inflamed) and the infected murine respiratory tract (Fig. S1). Alveolar macrophages are the dominant (60%–70%) CD11c^+^ cell subset in the normal lung, and are identified by Siglec F expression [19], [27] as well as by their high side scatter and high autofluorescence [28] resulting in an apparent moderate high levels of MHC class II expression (Fig. S1 A, B).

**Figure 1:**
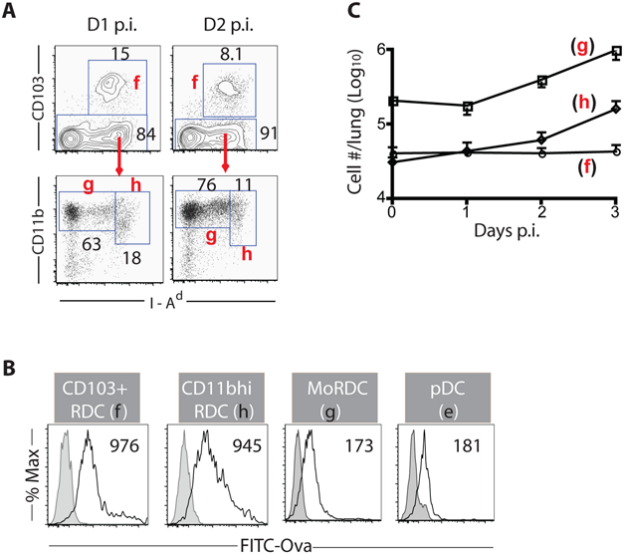
DC subsets in the normal and influenza-infected lung. (A and C) Conventional RDC subsets in the normal and influenza-infected lung. Hematopoietic origin cells in the lung suspensions are identified as CD45^+^. Among the CD45^+^CD11c^+^ cells, the highly auto-fluorescent alveolar macrophages (AM) and B220^+^ plasmacytoid DC are excluded from further analyses (see Fig. S1 A and B for gating and identification strategy). CD103^+^ RDC (f), MoRDC (g) and CD11b^hi^ RDC (h) in the uninfected (left) or infected (at d2 p.i., right) lung are identified (A) and enumerated (C) at indicated days p.i. (B) Ability of RDC subsets to take up soluble antigens *in vivo*. Mice are given i.n. FITC-labeled ovalbumin (FITC-Ova) or unlabeled Ova proteins 1 hr prior to sacrifice. The amounts (%) of FITC-Ova taken up by RDC subsets are compared to those of ova, and the numbers in the inserts indicate mean fluorescent intensity (MFI) of FITC-Ova (n>3).

We identified four subsets of DC among the CD11c^+^ Siglec F^−^ cells in the normal lung based on the expression level of MHC Class II and the presence of distinct cell surface markers. These include: 1) a small number of B220^+^Gr-1^+^MHC II^lo^ plasmacytoid DC (pDC) (Fig. S1A - panel e) and 3 more abundant RDC subsets, 2) CD103^+^MHC II^hi^CD11b^neg-hi^ DC (designated CD103^+^ RDC hereafter, Figs. 1A and S1A - panel f); 3) CD103^−^MHC II^hi^ CD11b^med-hi^ DC (designated CD11b^hi^ RDC, Figs. 1A and S1A - panel h); 4) CD103^−^MHC II^neg-med^ CD11b^hi^ monocytic DC (designated MoRDC, Figs. 1A and S1A - panel g). These latter 3 RDC subsets also differed in size, light scatters, and expression of various activation/differentiation markers (Fig. S1D and not depicted) reinforcing the view that the cells may be distinct RDC subsets.

Since the MoRDC are the predominant RDC subset, we considered this RDC subset to likely play a predominant role in antigen uptake and presentation in the respiratory tract particularly in response to a strong inflammatory stimulus such as virus infection. However, two subsets of RDC, the CD103^+^ RDC and CD11b^hi^ RDC were also potential candidates in antigen uptake and recognition because of their preferential localization to the airway mucosa of epithelium and the lung submucosa/parenchyma, respectively [19].

To monitor the relative efficiency with which these distinctive RDC subsets can take up antigen delivered into the respiratory tract, we use the standard approach of delivering fluorescein-tagged ovalbumin into the normal (uninfected) mouse respiratory tract by the intranasal (i.n.) route. Mice had been sacrificed and the uptake of this antigen by the four RDC subsets was evaluated by flow cytometry. As early as one hour post ovalbumin administration (Fig. 1B), uptake of the antigen by the RDC was demonstrable with the CD103^+^ RDC and the CD11b^hi^ RDC exhibiting the highest efficiency. As discussed below for migrant RDC in the draining lymph nodes, the tagged ovalbumin was localized in endocytic vesicles within the RDC as expected for immature DC surveying and taking up antigen at peripheral sites [1]. Of note, CD103^+^ and CD11b^hi^ RDC but not MoRDC or pDC, express high levels of costimulatory ligands and antigen presenting molecules (i.e., CD1d and MHC class II), and lack the co-inhibitory ligand B7-H1 (Fig. S1D and not depicted). These properties of the CD103^+^ and CD11b^hi^ RDC subsets would make them attractive candidates to serve as effective antigen presenting cells.

Following i.n. influenza (A/PR/8/34) infection, these RDC in the infected lungs retain the markers characteristic of the individual subsets at early times post infection, i.e., days 1–2 (Fig. 1A and S1D) and remain distinguishable at least up to d5 p.i. (not depicted). However, influenza infection results in a gradual but significant increase in the absolute number of CD11b^hi^ RDC and MoRDC infiltrating the lungs while the CD103^+^ RDC numbers are minimally increased (Fig. 1C).

### CD103^+^ and CD11b^hi^ DC subsets accumulate in the mediastinal lymph nodes after respiratory influenza virus infection

Distinct subsets of DC have been identified in the mediastinal lymph nodes (MLN) draining the murine lungs. RDC which have migrated to the MLN either constitutively from the normal (non-inflamed) lungs or in response to pulmonary inflammation/infection are believed to contribute to the DC population found in the MLN, but the contribution of the individual subsets of migrant RDC to the expanded population of DC found in the MLN following respiratory infection has not been clearly defined. We analyzed the DC populations liberated from the normal MLN and from the MLN undergoing sub-lethal influenza infection by two methods, mechanical disruption on or enzymatic digestion.

**Figure 2:**
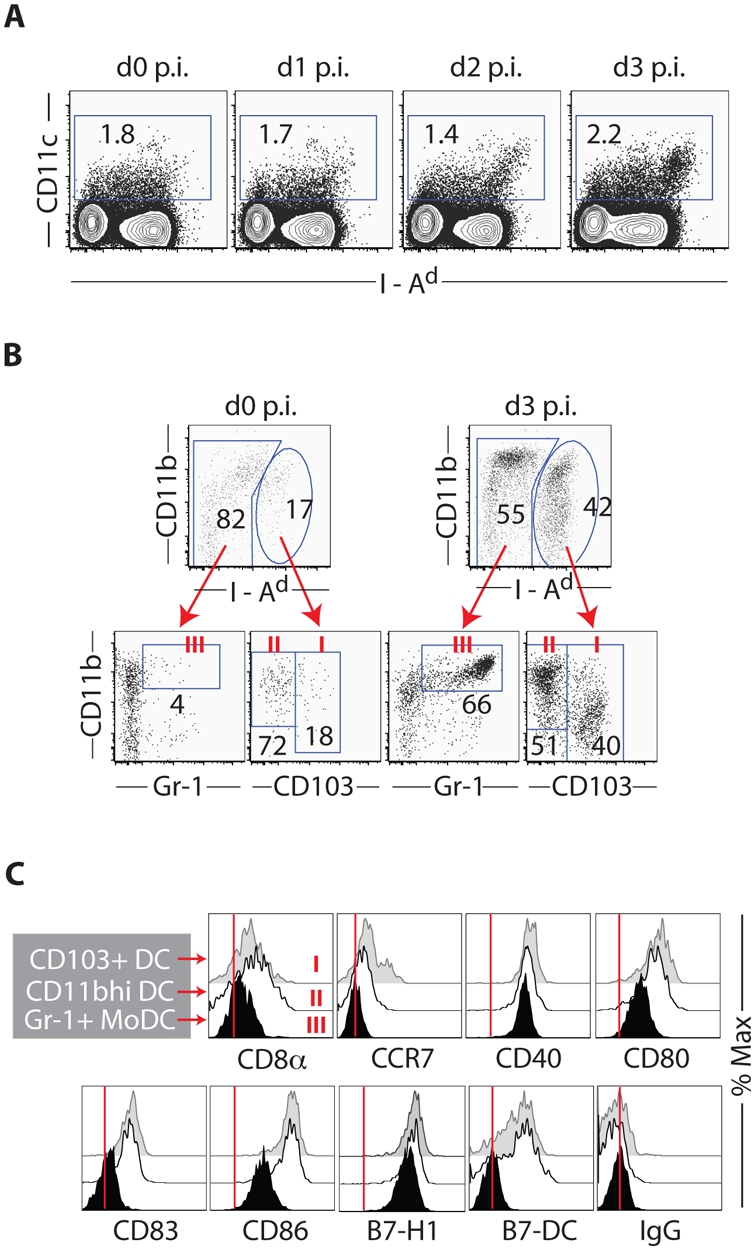
Progressive accumulation of CD103^+^ DC and CD11b^hi^ DC in the lung-draining lymph nodes of influenza-infected mice. (A) Accumulation of CD11c^hi^MHC II^hi^ DC in the MLN after influenza infection. Representatives of total CD11c^+^ cells in the MLN of influenza-infected mice at indicated days p.i. are shown. (B) Accumulation of three major DC subsets in the MLN of influenza-infected mice. CD103^+^ DC (CD11c^hi^MHC II^hi^CD103^+^CD11b^+/−^, group I), CD11b^hi^ DC (CD11c^hi^MHC II^hi^CD103^−^CD11b^hi^, group II) and Gr-1^+^ MoDC (CD11c^lo^MHC II^lo^CD103^−^Gr-1^+^CD11b^hi^, group III) are highly represented in the MLN at d3 p.i. (right panel) compared to those in uninfected animals (left panel) (see gating strategy in Fig. S2). (C) Phenotypic features of DC subsets in the MLN of influenza-infected mice at d3 p.i. Surface marker expression on CD103^+^ DC (Group I, gray), CD11b^hi^ DC (Group II, white) and Gr-1^+^ MoDC (Group III, black) identified in the MLN of infected (d3 p.i.) mice (right panel in B) is examined after staining with indicated mAbs. Vertical lines (|) indicate the median values of surface staining with isotype-matched control antibodies.

In the normal lymph node, DC displaying CD103^+^ or CD11b^hi^ phenotype are minimally represented and DC with the Gr-1^−^ MHC class II^int^ phenotype of MoDC are the dominant DC population by either method of lymph node cell dispersion (Fig. 2B and S2). Following enzymatic digestion [2], [7], however, a characteristic subset of MHC class II^+^ CD8αα^+^ node resident DC is also demonstrable among the CD11c^+^ cells in the lymph node (Fig. S3A). Following i.n. infection there is the expected increase [7] in the total number of CD11c^+^ cells in the MLN (Fig. 2A and S3A). Based on cell surface marker expression to distinguish DC subsets, CD103^+^ and CD11b^hi^ DC were readily demonstrable by d3 p.i. along with a Gr-1^+^ DC population expressing cell surface markers characteristics of inflammatory monocytic dendritic cells, i.e. Gr-1^+^ MoDC (Figs. 2B and S2). pDC numbers in the MLN increased only modestly over this period (not depicted). It is noteworthy that high-level expression of costimulatory ligands was largely restricted to the accumulating CD103^+^ and CD11b^hi^ DC and the chemokine receptor CCR7 was also selectively expressed only on these 2 DC subsets (Fig. 2C). By contrast, the CD8αα^+^ node resident DC likewise increased in number only modestly during the first 3 days of infection (Fig. S3A and not depicted) and while expressing highest levels of CD11c, this DC subset expressed lower levels of costimulatory ligands than aforementioned DC subsets (Fig. S3B). As observed for the lung resident RDC, these several DC subsets found in the MLN following infection had distinct size, cellular complexity and surface marker differences (Fig. S4) suggesting that they indeed represent distinct CD11c^+^ cell subsets.

The expression of high levels of MHC molecules and costimulatory ligands, characteristics of mature DC [1], by the CD103^+^ and CD11b^hi^ DC (Fig. 2C) would make them likely candidates to serve as potent APC for naïve virus-specific T cells. It is of interest that these two DC subsets in the draining MLN also express CD8α (and β) (Figs. 2C, S4A, and not depicted). However, unlike the lymph node-resident CD8αα^+^ which constitutively expressed this molecule (Fig. S4A), companion studies analyzing the lymph node DC subsets in infected T lymphocytes deficient Rag1^−/−^ mice suggest that the CD8α displayed by these two MHC II^hi^ DC subsets is most likely acquired as CD8α/β heterodimers from T cells within the draining MLN via trogocytosis ([29], [30], and not depicted).

### Source of the CD103^+^ and CD11b^hi^ DC subsets in the draining MLN during respiratory influenza virus infection

**Figure 3:**
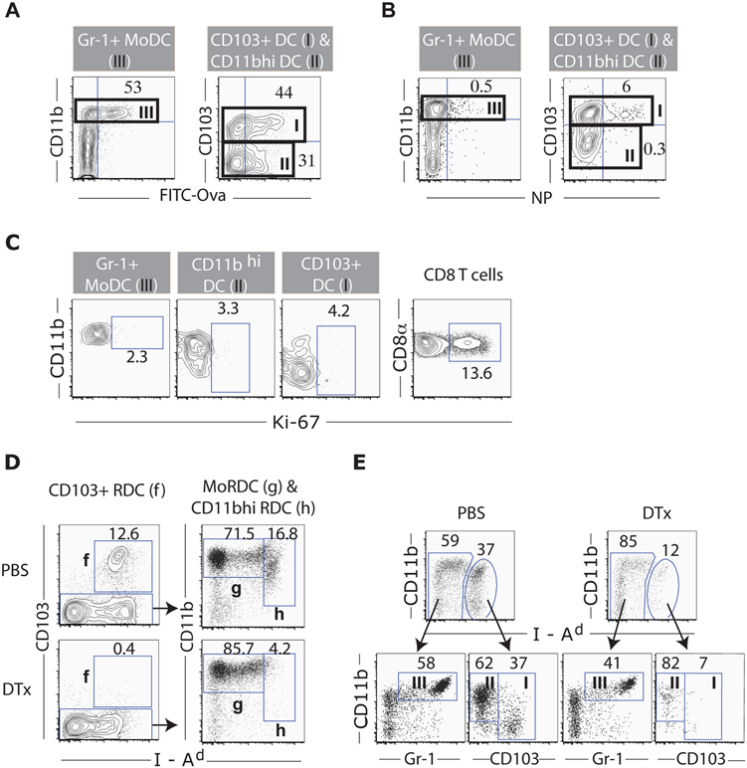
Sources of DC subsets in the MLN of influenza-infected mice. (A and B) Acquisition and transportation of FITC-Ova (A) or viral proteins (B) by lung-residing DC into the MLN of infected mice. (A) Fluorescent (FITC)-tagged or unlabeled Ova proteins were i.n. instilled into influenza-infected mice at d2 p.i. One day later, DC subsets in the MLN of the Ova-instilled infected mice are examined for the uptake (%) of FITC-Ova. (B) The specific % NP^+^ DC subsets in the MLN of infected mice at d3 p.i. is examined after intracellular staining for viral nucleoprotein (NP). (C) Examination of local proliferation of DC subsets in the MLN of infected mice. DC isolated from the MLN of i.n. infected mice at d3 p.i. are intracellularly stained for an nuclear antigen Ki-67. Ki-67 staining on CD8^+^ T cells isolated from MLN of infected mice at d7 p.i. are depicted as a positive control. (D and E) Accumulation of CD11b^hi^ DC and CD103^+^ DC in the MLN of infected mice requires the presence of the corresponding counterparts in the lung. (D) Naïve DTR mice are instilled i.n. with DTx (lower panel) or vehicle alone (top panel), and depletion of the CD11c^+^ RDC subsets in the lung is examined 48 hr later (n>5). (E) DC subsets in the MLN of influenza-infected mice are identified at d3 p.i. in the DTR mice i.n. given either DTx or PBS one day prior to infection (n>10).

Since the MHC II^hi^ CD103^+^ and CD11b^hi^ RDC as well as the MoRDC were present in both the normal and infected lungs, the accumulation of DC with the same phenotypes in the MLN draining infected lungs most likely reflected the accelerated migration of these DC subsets from the lungs to the draining nodes in response to infection rather than recruitment of DC progenitors from the bone marrow to the MLN or the *de novo* differentiation/maturation of CD103^−^CD11b^lo/med^ LN-resident DC into these respective DC subsets upon infection. To determine a potential link between the lung-residing RDC and these MHC II^hi^ DC accumulating in the MLN during infection, we first examined the effect of i.n. administration of soluble FITC-Ova at d2 p.i. on fluorescenated protein displayed by DC subsets in the MLN 24 hr later. We chose this time frame for labeled antigen administration and cell analysis because this time frame coincided with the rapid accumulation of these two MHC II^hi^ DC subsets in the MLN (see below). Therefore, over this 24 hr time frame there would be sufficient time for cells in the infected lungs including RDC subsets to take up antigen and then migrate from infected lung to the MLN. We found that the two MHC II^hi^ DC subsets, CD103^+^ (44%, Fig. 3A - I) and CD11b^hi^ (31%, Fig. 3A - II) DC, and the Gr-1^+^ MoDC (53%, Fig. 3A - III) were FITC^+^, consistent with the likely lung-origin of these DC populations. Of note, confocal images of FITC-Ova^+^ cells in the MLN revealed that FITC-Ova protein is localized within intracellular organells (Fig. S5), indicative of endo/phagocytosis, rather than diffuse cell surface binding, and therefore consistent with the concept that FITC-Ova was directly taken up by RDC via endo/phagocytosis prior to their migration into the MLN from the lungs.

In companion experiments we determined whether any of these DC populations found in the MLN displays intracellular expression of influenza nucleoprotein (NP) by flow-based analysis. This analysis revealed that CD103^+^ DC were the predominant infected - NP expressing - cell type in the MLN (Fig. 3B). This is consistent with our recently published findings [31] that the CD103^+^ RDC subset is most susceptible to influenza infection *in vivo* and *in vitro*. Although the diffuse intracellular pattern (cytoplasmic as well as nuclear) of influenza NP expression in the CD103^+^ DC from the MLN at d3 p.i. mimics expression profile of this viral protein in CD103^+^ RDC infected directly *ex vivo* (Fig. S5), NP^+^ cells represented only a fraction of the CD103^+^ DC isolated from the draining nodes. We cannot exclude the possibility that the majority of the CD103^+^ DC in the MLN may have acquired influenza viral antigens including NP from infected cells in the respiratory tract prior to the migration of the RDC into the draining nodes. We further determined that the accumulation of three major DC subsets and in particular the MHC II^hi^ CD103^+^ and CD11b^hi^ DC in the MLN of infected animals was not due to recent local cell proliferation, as less than 5% of these DC subsets had undergone recent cell division as determined by the expression of the nuclear antigen Ki-67, a marker for recent cell proliferation [32] (Fig. 3C); and administration of the thymidine analog BrdU continuously during the first 3 days of infection resulted in a fraction (<30%) of CD103^+^ and CD11b^hi^ DC incorporating this analog into DNA (Fig. S6A).

To more directly determine the contribution of the lung-residing CD103^+^ and CD11b^hi^ RDC subsets to the corresponding DC subsets accumulating in the MLN in response to respiratory virus infection, we exploited the transgenic (tg) mice expressing the non-human primate diphtheria toxin (DTx) receptor (DTR) driven off the murine CD11c promoter [33]. Administration of DTx to the DTR mice results in a toxin dose- and CD11c expression level-dependent depletion of CD11c^+^ DC with CD11c^hi^ cells most sensitive to the lowest toxin doses. We found that the local (i.n.) administration of the low dose of DTx to DTR mice immediately prior to i.n. influenza infection resulted in the preferential elimination of the two CD11c^hi^ DC subsets, the MHC II^hi^ CD103^+^ and CD11b^hi^ RDC from the respiratory tract (Fig. 3D) and also inhibited the accumulation of the CD103^+^ and CD11b^hi^ DC subsets in the MLN after infection, with only a modest effect on Gr-1^+^ MoDC accumulation (Fig. 3E and Fig. S6B). Of note, high dose i.n. DTx administration resulted in the depletion of these two CD11c^hi^ RDC subsets as well as MoRDC from the lungs of the DTR mice and also partial depletion of LN DC from the MLN (not depicted).

These findings, suggesting the lung origin of the CD103^+^ and CD11b^hi^ DC subsets accumulating in the MLN, were further supported by studies employing CCR7^−/−^ mice. The chemokine receptor CCR7 plays an essential role in the migration of DC from peripheral sites to lymphoid organs [5], [6] and is expressed by the CD103^+^ and CD11b^hi^ DC subsets, but not the Gr-1^+^ MoDC in the draining MLN of infected mice (Fig. 2C). CCR7^−/−^ mice exhibit a normal component of lung-residing CD103^+^ and CD11b^hi^ RDC ([34] and not depicted) but these two DC subsets (unlike Gr-1^+^ MoDC) failed to accumulate in the MLN of influenza-infected CCR7-deficient mice (Fig. S6 C, D). Taken together, these data suggest that the accumulation of CD103^+^ and CD11b^hi^ DC in the MLN of influenza-infected mice results largely, if not exclusively, from infection-induced migration of their counterparts present in the lungs rather than through recruitment of circulating DC progenitors or proliferative expansion/conversion of resident cells in the MLN.

### Tempo of migrant respiratory DC accumulation and antigen transport into the MLN during respiratory viral infection

**Figure 4:**
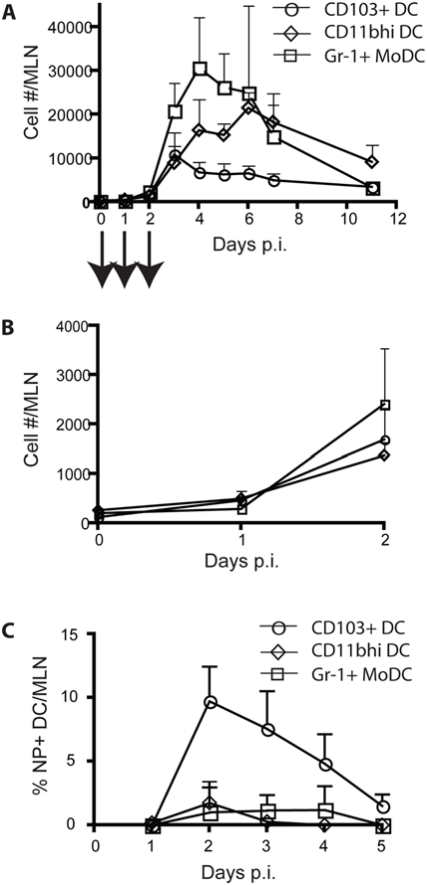
Kinetics of the accumulation of three major DC subsets in the MLN of influenza-infected mice. (A and B) Mice were i.n. infected with influenza, and at various days p.i., Gr-1^+^ MoDC (□), CD11b^hi^ DC (⋄) and CD103^+^ DC (xˆ) in the MLN of the infected mice are identified (as shown in Fig. S2) and enumerated (A). The magnitude of DC subsets accumulated in the MLN from d0–2 p.i. in (A) is expanded in (B) (mean±SD; n = 4–8 mice/group). (C) Kinetics of the accumulation of NP^+^ DC subsets in the MLN of influenza-infected mice from d1 to d5 p.i. The % NP^+^ DC subsets – Gr-1^+^ MoDC (□), CD11b^hi^ DC (⋄), and CD103^+^ DC (xˆ) – are represented after subtracting that of non-specific staining with an irrelevant mAb (mean±SD; n = 4–5 mice/group).

In view of the above findings implicating the lung as the site of origin of the CD103^+^ and CD11b^hi^ DC subsets accumulating in the draining MLN of influenza-infected mice (as well as at least some of the Gr-1^+^ MoDC in the MLN), it was of interest to examine the kinetics of accumulation of these distinct DC subsets in the MLN, as a basis for establishing if one or more of these DC subsets may play a critical role in antigen delivery and/or presentation to adaptive immune cells responding in the MLN (see below). To this end, we enumerated the number of each of these 3 DC subsets present in the MLN before and at various times after i.n. influenza virus infection. As observed in Figure 4 A and B, the time course of DC accumulation in the MLN differs for each DC subsets. While the initial number of each of these DC subsets in the MLN increase between d1–2 p.i. (Fig. 4B), they differ in their days of peak accumulation with CD103^+^ and CD11b^hi^ DC reaching a peak at d3 and 6 p.i., respectively. The latter accumulate in the MLN at higher magnitude, reminiscent of the tempo of CD103^+^ and CD11b^hi^ DC accumulating in the lungs at an early infection (d 1–3 p.i., Fig. 1C). The numbers of these two DC subsets progressively decrease after peak accumulation, but remain at a relatively high level by 1.5–2 wks p.i. On the other hand, a more robust accumulation of Gr-1^+^ MoDC with faster tempo, peaking at d4 p.i., was observed. Following a gradual decrease after their peak again, the Gr-1^+^ MoDC are minimally detectable by 2 wks p.i. We also examine the kinetics of expression of the influenza NP by these three DC subsets in the MLN (Fig. 4C). NP^+^ cells were largely restricted to this CD103^+^ DC subset and expression of the NP by the CD103^+^ DC subset was most prominent early in infection, i.e. greatest at d2 p.i. NP^+^ DC were not detectable in the MLN after day 5 of infection (not depicted).

### CD103^+^ and CD11b^hi^ RDC are required for the activation of antigen-specific naïve CD8^+^ T cells in the MLN

The Gr-1^+^ MoRDC were the most abundant RDC population both in the influenza infected lungs and in the draining the MLN, and would therefore be expected to play the most prominent role in the activation of antigen-specific T cells in the MLN through viral antigen delivery to DC resident in the MLN and/or by acting directly as APC. However, because they expressed high levels of MHC molecules and costimulatory ligands as well as their ability to take up antigen in the infected respiratory tract, the CD103^+^ and CD11b^hi^ RDC also had the potential to serve as APC after their migration to the draining MLN (Fig. 2C). To determine the contribution of lung-resident CD103^+^ and/or CD11b^hi^ RDC to the induction of an *in vivo* adaptive immune response in the draining MLN following influenza infection, we examined the impact of elimination of CD103^+^ and CD11b^hi^ RDC from the respiratory tract on the activation and proliferation of virus-specific naïve CD8^+^ T cells in the DTR model.

**Figure 5:**
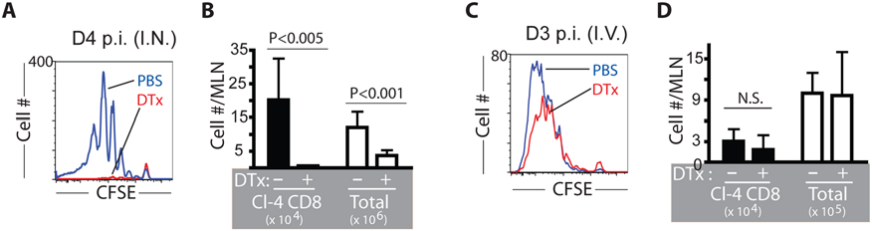
The CD11b^hi^ and CD103^+^ RDC subsets are required for the induction of optimal proliferative expansion of virus-specific CD8^+^ T cells responding to respiratory influenza virus infection *in vivo*. (A–D) Ablation of both lung-residing CD11b^hi^ and CD103^+^ DC subsets prior to infection resulted in a near absence of virus-specific naïve CL-4 CD8^+^ T cell proliferation in response to respiratory (A and B), but not intravenous (C and D), influenza virus infection. Following CFSE-labeled CL-4 CD8^+^ T cell transfer into DTR mice, DTx (or PBS) is administered i.n. one day prior to infection. Four (i.n.) or 3 (i.v.) days after virus infection, the division profiles of CL-4 T cells (A or C) and total recovery of the number of CL-4 T cells (left in B or D) and total cellularity (right in B or D) in the MLN of infected DTR mice were determined (mean±SD; n = 4–6).

Naïve CSFE dye-labeled influenza hemagglutinin (HA) specific Clone 4 (CL-4) TCR tg CD8^+^ T cells were transferred into DTR recipients, followed sequentially at 24 hr intervals by low dose i.n. administration of DTx to selectively eliminate these two RDC subsets and then i.n. influenza infection. As reported previously [8], [35], and demonstrated here (Fig. S7), adoptively transferred CSFE-labeled CL-4 T cells vigorously proliferate in the MLN between days 3 and 4 of infection of wild type control (PBS treated) DTR mice (Fig. 5 A, B). By contrast, ablation of these 2 CD11c^hi^ MHC II^hi^ RDC subsets results in the near complete absence of CD8^+^ T cell proliferation/expansion in the draining MLN (Fig. 5 A, B). Low dose i.n. DTx administration did not adversely affect the antigen presenting capacity of DC in the draining MLN, as the naïve CL-4 T cells vigorously proliferated in the MLN of DTx-treated DTR recipients infected with influenza virus by the intravenous route (Fig. 5 C, D), and DTx administration into the respiratory tract did not significantly affect the number of total CD11c^+^ cells in the MLN when examined at 48 hr post DTx treatment (not depicted).

The critical role of one or both of these migrant RDC subsets in T cell activation was further substantiated in studies employing dye-labeled CCR7^+/+^ CL-4 T cells transferred into CCR7^−/−^ recipients. After i.n. infection, there was a markedly reduced proliferative expansion of the CL-4 T cells in the MLN of the CCR7-deficient mice (Fig. S8A) whose lung-residing CD103^+^ and CD11b^hi^ DC are unable to do emigrate out of the respiratory tract in response to virus infection (Fig. S6 C, D). Since the architecture of secondary lymphoid organs, e.g. lymph nodes, is abnormal in CCR7-deficient mice, the reduced CD8^+^ T cell proliferation in the MLN of these animals could result from a defect in the interaction between naïve T cells and lymph node-resident DC. To assess this possibility we examine the capacity of the naïve TCR transgenic CD8^+^ T cells to respond in the MLN after i.v. administration of infectious influenza virus. Influenza infection by the intravenous route did not affect proliferative expansion of the CCR7-competent CL-4 CD8^+^ T cells in the MLN of the CCR7-deficient host (Fig. S8B), suggesting that the absence of the CCR7 receptors does not alter the ability of LNDC to interact with and activate naïve CD8^+^ T cells, at least under conditions of intravenous virus infection.

### Migrant CD103^+^ and CD11b^hi^ RDC in the MLN differ in their ability to stimulate the activation and differentiation of naïve virus-specific CD8^+^ T cells into effector T cells

The above findings implicated one or both of the two migrant RDC subsets found in the MLN after respiratory virus infection as critical for the induction of anti-viral T cell responses presumably through their capacity to transport the viral antigen to the draining MLN and possibly to directly serve as APC for naïve T cells. To better define the role of these migrant RDC subsets as well as the other DC subsets found in the MLN after infection in the antigen presentation to naïve T cells, we first isolated (after mechanical lymph node disruption) the major CD11c^+^ DC populations (i.e., primarily of CD103^+^ DC, CD11b^hi^ DC, Gr-1^+^ MoDC and a smaller number of B220^+^ pDC) from the MLN of influenza-infected mice at d3 p.i. (Fig. S9). We co-cultured these DC subsets with CSFE-labeled influenza HA-specific naïve TCR tg CD8^+^ or CD4^+^ T cells to evaluate the capacity of these LN-derived DC subsets to drive naïve T cell activation/differentiation.

**Figure 6:**
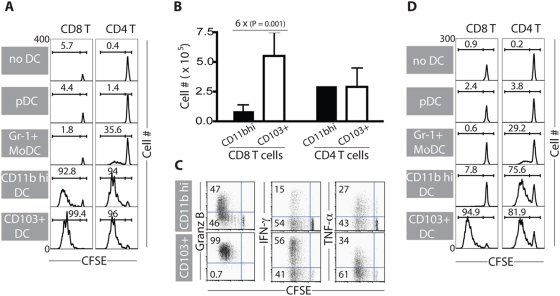
Airway-derived migrant CD103^+^ DC in the inflamed MLN are superior in presenting or cross-presenting influenza virus antigens to naïve CD8^+^ T cells. (A–C) Efficiency of DC subsets in the MLN of infected animals to stimulate naïve either CD8^+^ or CD4^+^ T cells after intranasal infection with infectious influenza virus. DC subsets isolated from the MLN of mice infected 3 d earlier with influenza virus were co-cultured with CFSE-labeled HA-specific naïve TCR tg CD8^+^ or CD4^+^ T cells for 4 days. Proliferation profile (A) or total recovery (B) of viable CD8^+^ or CD4^+^ T cells stimulated by the MLN-derived DC subsets are shown (mean±SD; n = 5). Effector functions of CD8^+^ T lymphocytes stimulated by either CD11b^hi^ DC or CD103^+^ DC are examined at d4 co-culture (C). (D) Efficiency of DC subsets in the MLN to stimulate naïve CD8^+^ or CD4^+^ T cells after intranasal administration of non-infectious influenza virions. DC subsets isolated from the MLN of mice inoculated 3 d earlier with inactivated influenza virus were co-cultured with CFSE-labeled HA-specific naïve TCR tg CD8^+^ or CD4^+^ T cells for 4 days. Representatives of proliferation profile of T cell subsets are depicted (n = 3).

We found that B220^+^ pDC fail to activate either naïve CD4^+^ or CD8^+^ T cells. By contrast, the dominant Gr-1^+^ MoDC could stimulate naïve CD4^+^ T cells, albeit to a modest extent, and CD8^+^ T cells minimally (Fig. 6A). However, both pDC and Gr-1^+^ MoDC could trigger tg CD8^+^ T cell proliferation when pulsed with the pre-processed epitope peptide (Fig. S10). By contrast, either CD103^+^ or CD11b^hi^ DC isolated from the MLN of infected mice could support CD4^+^ T cell activation, proliferative expansion (Fig. 6 A, B) and differentiation into effector cytokine-producing cells (Fig. S11) with comparable efficiency. These two DC subsets, however, did differ in their ability to trigger naïve CD8^+^ T cell activation and expansion. While both DC subsets could activate naïve CD8^+^ T cells, the CD103^+^ DC activated a larger fraction of the naïve CD8^+^ T cells (Fig. 6A) and triggered more extensive expansion and increased recovery of responding T cells than the CD11b^hi^ DC (Fig. 6B). This difference in the efficiency of naïve CD8^+^ T cell stimulation by the two DC subsets was also evident when the differentiation state of the responding T cells was examined. The expression of the cytolytic T lymphocyte effector molecules, granzyme B and interferon-γ, but TNF-α, were preferentially elevated in responding CD8^+^ T cells stimulated by the CD103^+^ DC (Fig. 6C).

CD8αα^+^ LNDC have been previously reported to be an important APC for stimulation of influenza-specific naïve CD8^+^ T cells through the uptake and presentation of viral antigen delivered to the MLN by migrant RDC [7]. Elimination of the CD103^+^ and CD11b^hi^ RDC from the respiratory tract (by i.n. DTx administration) or the inability of these RDC to migrate (CCR7 deficiency) to the MLN would result in the failure of viral antigen delivery to the MLN and as of consequence the inability of CD8αα^+^ LNDC to capture viral antigen and activate naïve CD8^+^ T cells. When the CD8αα^+^ LNDC were isolated from the draining MLN at d3 p.i. and separated from the CD103^+^ and CD11b^hi^ migrant RDC (also CD8α^+^, albeit at a lower level), compared to the two migrant CD11c^hi^ MHC II^hi^ subsets, this LNDC subset exhibited only modest antigen presenting activity comparable to that of the migrant Gr-1^+^ MoDC (Fig. S12A), but could present when peptide pulsed (Fig. S12B).

Certain DC subsets appear to be specialized to take up non-infectious antigenic materials, process and present such antigens along the MHC class I presentation pathway to naïve CD8^+^ T cells via ‘cross-presentation’ [36], [37], [38]. Cross-presentation has been implicated as the mechanism to allow antigen, such as soluble proteins, as well as viruses, which do not infect DC, and tumor cell antigens to stimulate CD8^+^ T cell responses [38]. To investigate cross-presentation of influenza virus by the migrant RDC we introduce non-infectious intact influenza virion vaccine into the lungs by the i.n. route and examined the capacity of the DC subsets isolated from the MLN of vaccine treated at donors to stimulate directly *ex vivo* virus-specific naïve CD4^+^ and CD8^+^ T cells. We found that, similar to infectious virus, non-infectious virions were processed and presented to naïve CD4^+^ T cells by the CD11b^hi^ DC and the CD103^+^ DC with comparable efficiency (Fig. 6D). By contrast, the CD103^+^ DC preferentially (if not exclusively) presented peptides derived from *in vivo* airway processing of non-infectious virions to naïve CD8^+^ T cells and trigger T cell proliferation (Fig. 6D).

## Discussion

DC found at peripheral body sites such as mucosal surfaces or the skin are not a uniform population, but rather exist as distinct cell subsets which differ in the expression of certain cell surface markers. While the functional significance of these DC subset differences is poorly understood, different DC subsets are believed to play distinct roles in the induction and expression of innate and adaptive immune responses [1], [39]. In this report, we demonstrate that two distinct subsets of DC in the murine respiratory tract displaying the CD103^+^CD11b^+/−^ (CD103^+^) and the CD103^−^CD11b^hi^ (CD11b^hi^) cell surface phenotypes are the primary antigen presenting cells, which take up viral antigen and migrate to the draining MLN to initiate the induction of the adaptive T cell responses to influenza virus infection. Interestingly, while both RDC subsets can stimulate influenza specific CD4^+^ T cell responses with comparable efficiency, only the migrant CD103^+^ RDC subset (when isolated from the MLN of virus infected animals) is capable of driving extensive proliferation and full differentiation of naïve antigen specific CD8^+^ T cells into effector cells. Importantly, this CD103^+^ RDC subset is the only cell type capable of efficiently capturing non-infectious influenza virions administered into the respiratory tract and presenting *in vivo* processed antigen to naïve influenza specific CD8^+^ T cells in the MLN. Thus, this RDC subset may be unique in its capacity to carrying out “cross-presentation” to CD8+ T cells of non-infectious (particulate and soluble) antigens entering the respiratory tract and viral antigen from virally infected cells in the respiratory tract.

As demonstrated here and elsewhere [18], [19], [40], several distinct subsets of the RDC (as defined by cell surface marker expression) can be identified in the normal/non-inflamed murine respiratory tract. Two of these subsets, the CD103^+^ and CD11b^hi^ DC, are minimally represented in the LN draining the lower respiratory tract, but CD103^+^ and CD11b^hi^ DC did accumulate significantly in the MLN following i.n. influenza infection. The tempo of the CD103^+^ and CD11b^hi^ DC accumulation, i.e. onset between 1 and 2 days p.i., paralleled the previously reported tempo of activation of naïve CD8^+^ T cell activation in the MLN following sub-lethal influenza infection [7], [8] suggesting a potential role for these accumulating CD103^+^ and CD11b^hi^ DC in T cell activation in the MLN. At least 3 mechanisms could account for the accumulation of these DC subsets in the MLN in response to respiratory infections; 1. migration of CD103^+^ and CD11b^hi^ RDC from the respiratory tract to the MLN in response to infection; 2. mobilization of blood-borne DC progenitors from the circulation into the MLN in response to infection; 3. acquisition of these cell surface markers by LN-resident DC in response to infection.

Multiple lines of evidence described in this report suggest that the CD103^+^ and CD11b^hi^ DC accumulating in the MLN are indeed migrant RDC in the respiratory tract in response to virus infection. Along with standard techniques of DC detection in the MLN after uptake of dye and/or labeled antigen introduced into the respiratory tract [7], [23], [41] and evaluation of recent cell division by BrdU uptake and Ki- 67 expression, which argued for terminally differentiated cells of respiratory tract origin as a source of these DC accumulating in the MLN, we were unable to inhibit the accumulation of these 2 DC subsets in the MLN by blocking CD62L (not depicted) - a critical adhesion molecule for DC migration from the circulation into secondary lymphoid organs [9], [11], [42]. More importantly, we found that in DTR mice the selective ablation of the CD11c^hi^ CD103^+^ and CD11b^hi^ RDC subsets by low dose DTx administration to the respiratory tract at the time of respiratory virus infection selectively inhibited the accumulation of CD103^+^ and CD11b^hi^ DC in the MLN after infection. Consistent with the result in DTR mice, CCR7^−/−^ mice, which have a normal component of the CD103^+^ and CD11b^hi^ DC in the respiratory tract and require interaction between CCR7 on activated DC with its chemokine ligands for efficient migration to the DLN [5], [34], [43], also failed to accumulate the CD103^+^ and CD11b^hi^ DC in the inflamed MLN. In this connection, it is particularly noteworthy that the substantially diminished migration of the CD103^+^ and CD11b^hi^ RDC to the MLN in response to respiratory virus infection as a result of depletion (DTx treatment) or absence of a response to a chemotactic stimulus (CCR7^−/−^ mice) also resulted in markedly diminished naïve CD8^+^ T cell activation in the MLN.

Although numerous studies have examined the impact of introduction of inert antigens and infectious agents into the respiratory tract on respiratory and LN DC properties and function in the induction of adaptive immune responses [7], [41], [44], [45], [46], the relative contribution of respiratory tract-derived and LN-resident DC in driving T cell responses to respiratory tract antigens or pathogens has been difficult to define. Indeed, there is compelling evidence that migrant RDC may function primarily in antigen transport and delivery to LN-resident DC in the draining LN which in turn are largely responsible for induction of adaptive T cell immune responses [22], [41], [44], [45]. Our findings on the effect of DTx ablation of RDC subsets and the impact of CCR7 deficiency on DC accumulation and T cell activation in the MLN could simply reflect the role of migrant CD11c^hi^ CD103^+^ and CD11b^hi^ RDC in antigen transport. However, direct analysis of the capacity of DC subsets isolated from the MLN of infected animals at the time of naive CD8^+^ T cell activation (d3 p.i.) to support naïve CD8^+^ and CD4^+^ T cell activation reveals that the migrant CD103^+^ and CD11b^hi^ RDC isolated from the MLN are most efficient at stimulating naïve T cells. The migrant Gr-1^+^ MoRDC are much less efficient on a per cell basis at stimulating naive T cells, particularly naïve CD8^+^ T cells. The cells are, however, the major migrant RDC subset entering the draining MLN and can stimulate naïve CD4^+^ T cells with modest efficiency. Although Gr-1^+^ MoDC had been implicated as important APC for naïve T cell activation in several models [12], [13], [14], our findings on the impact of low dose i.n. DTx administration (as well as CCR7 deficiency) on the numbers and migration of these inflammatory DC to the MLN during infection suggest a minor role for these cells as APC at least in the respiratory tract during natural virus infection.

Our ability to liberate the CD8αα^+^ LNDC from the MLN in significant numbers is critically dependent on the method of LN disruption and DC isolation. Using enzymatic treatment of the disrupted MLN, we isolated sufficient numbers of these cells from the d3 p.i. draining MLN to examine their antigen presenting activity for naïve CD8^+^ T cells with minimal contamination by the two other CD8α^+^ DC subsets in the MLN (i.e., the migrant CD103^+^ and CD11b^hi^ RDC). We found that similar to migrant Gr-1^+^ MoDC the CD8αα^+^ LNDC had modest antigen presenting capacity. We cannot exclude the possibility that a fraction of the LN CD8αα^+^ DC upregulate MHC II at d3 p.i. and are including among the sorted population of MHC II^hi^ migrant RDC. Thus this subpopulation of CD8αα^+^ DC could have potent antigen presenting capacity like the migrant CD11b^hi^ RDC. Although our results do not exclude an important role of CD8αα^+^ LNDC in antigen presentation to naïve T cells as shown by Belz et al. [7], our findings suggest a major role for migrant RDC, especially CD103^+^ RDC, as the primary APC for naïve CD8 T cells responding to respiratory virus infection. The aforementioned study of Belz et. al. in the influenza model demonstrating both LN-resident CD8αα DC as well as migrant DC serving as APC in response to flu infection did not, however, identify the nature of migrant RDC. Our study does not exclude the possibility that either or both subsets of migrant RDC – CD103^+^ and CD11b^hi^ – could transfer viral antigen to the LN-resident DC upon migration into the MLN. Although we did not observe an efficient antigen presentation by lymph node-resident CD8αα^+^ DC following influenza infection, factors such as virus strain, inoculum dose, method of DC isolation, undoubtedly could explain differences in the phenotype and APC activity of the DC observed by us and other investigators. It is noteworthy that our findings also complement a recent report [47] in which Langerin (Lg)^+^ RDC were shown to contribute to the CD8 T cell response to influenza infection. We and others have observed that CD103^+^ RDC are also Lg^+^ [19], [48] (T. Kim and T. Braciale, unpublished observation). Furthermore, our study suggests that the CD11b^hi^ RDC not only serve as potent APC for naïve anti- viral CD4^+^ T cells but also may contribute to the induction of anti- viral CD8^+^ T cell responses albeit with a lower efficiency. How these two subsets of RDC interact to orchestrate CD8/CD4 T cell response to respiratory viral infection remains to be determined.

The CD103^+^ and CD11b^hi^ RDC differ not only in their anatomic localization, but also notably in their respective capacity to present viral antigen to naïve CD8^+^ T cells. This was most clearly demonstrated by our observation that only the CD103^+^ RDC subset in the MLN can efficiently capture, process and present to naïve CD8^+^ T cells non-infectious viral antigen (virions) introduced into the respiratory tract. This result was unexpected as this RDC subset in the lung does not express markers linked to DC specialized for cross-presentation, e.g. CD8α [25], [26] or mannose receptor [49], not depicted). However, the intimate association of the CD103^+^ RDC subset with the mucosal epithelium [19] would make these cells ideally suited to preferentially sample non-replicating (soluble or particulate) antigens introduced onto the mucosal epithelial surface.

We were able to detect expression of influenza NP in the migrant RDC isolated from the MLN of infected mice suggesting that a fraction of the migrant RDC found in the MLN were virally infected. As reported by us for RDC isolated from the uninfected lungs [31] the migrant CD103^+^ RDC subset was most susceptible to infection. As noted above migrant CD103^+^ RDC were more efficient than CD11b^hi^ RDC in triggering CD8^+^ T cell activation/proliferation and in driving effector T cell differentiation during virus infection. We cannot, however, conclude that this increased effectiveness of the CD103^+^ RDC as APC is directly linked to their infectibility, i.e. that direct infection of the RDC enhances their potency as APC for naïve CD8^+^ T cells. Only a small fraction of the CD103^+^ RDC in the nodes are infected. Since this DC subset appears to pick up, process and present exogenous viral antigen (Fig. 6D) to naïve CD8^+^ T cells, the majority of the migrant CD103^+^ RDC, which are NP^−^, probably (or indeed likely) function as APC by uptake of viral antigen from virally infected cells in the infected lungs prior to the migration to the MLN. Similarly the ability of the CD103^+^ RDC to preferentially stimulates naïve CD8^+^ T cells is unlikely to be directly related to the localization of this RDC subset in the respiratory tract and their accessibility of viral antigen, since both the CD103^+^ and CD11b^hi^ RDC can capture viral antigen produced by either natural infection or vaccine installation in the respiratory tract and efficiently present *in vivo* processed antigen to naive CD4^+^ T cells. Rather, the higher efficiency of the CD103^+^ RDC subset to cross-present particulate viral antigen vaccine or (as recently reported) soluble proteins introduced into the respiratory tract [22] as well as viral antigen produced during natural infection most likely reflects elevated expression of components of the class I presentation pathway selectively by CD103^+^ RDC and/or unique adaptation of this RDC subset for the uptake, processing and delivery of processed antigen to MHC class I molecules [49], [50] (T. Kim and T. Braciale, Manuscript in preparation). While differences in the efficiency of processed peptide/MHC I complex formation and consequently the strength of signal delivered to the TCR by these 2 RDC subsets could explain the ability of the CD103^+^ RDC to more efficiently drive the development of CD8^+^ T cell effector function, we believe that the CD103^+^ and CD11b^hi^ RDC subsets may also differ in their capacity to deliver accessory signals necessary to drive CD8^+^ T effector cell differentiation.

The CD103^+^ and CD11b^hi^ RDC continued to increase in numbers in the MLN for up to 5 days p.i. In parallel, there was a corresponding large influx of Gr-1^+^ MoDC. These CD11c^lo^F4/80^+^MHC II^lo^ DC are present at the minimal levels in the normal respiratory tract and MLN. They presumably arose from circulating Gr-1^+^ monocyte progenitors which entered the respiratory tract in response to infection. These Gr-1^+^ MoDC which, as demonstrated here, can capture antigens and then migrate by a CCR7-independent mechanism to the MLN have been reported to act as APC [12], [13], [14], [51]. These migrant Gr-1^+^ MoDC in the MLN appear, however, to have minimal capacity to present viral antigen to T cells and only to CD4^+^ T cells. Elevated numbers of all 3 of these migrant RDC populations are detectable in the MLN out to at least d10 p.i. Since the capacity of DC in the MLN to stimulate naïve T cells is markedly diminished by d10 p.i. [7] (T. Kim and T. Braciale, Manuscript in preparation), the reason for the elevated numbers of these migrant RDC in the MLN after virus clearance [52] remains to be determined, although they may play a role as APC in memory T cell generation [53], [54] (Kim and Braciale, Manuscript in preparation).

In conclusion, we have demonstrated that several distinct subsets of RDC migrate to the LN draining the respiratory tract in response to influenza virus infection. Our results indicate that antigen presenting capacity resides predominantly, if not exclusively, with the migrant RDC and only 2 of these migrant RDC subsets (i.e., the CD103^+^ and CD11b^hi^ DC) can efficiently activate naïve antigen-specific T cells in the MLN in response to infection. The ability of the CD103^+^ RDC subset to preferentially present antigen to CD8^+^ T cells both during infection and after viral vaccine instillation into the respiratory tract, along with the localization of this RDC subset within the airway mucosa, makes this DC subset a potentially important candidate cell type for further analysis in the study of respiratory disease pathogenesis and in mucosal vaccine development.

## Materials and Methods

### Mice

Female BALB/c mice (H-2^d^) were purchased from the Taconic Farms (Germantown, NY). Transgenic (Tg) mice expressing non-human primate Diphtheria Toxin Receptor (DTR) under the control of the murine CD11c-promoter (C.FVB-Tg *^Itgax-DTR/EGFP^* 57Lan/J, H-2^d^) mice and Thy-1.1 Clone 4 (Cl-4) TCR Tg mice (H-2^d^) were obtained from The Jackson Laboratory (Bar Harbor, ME). Thy-1.2 Cl-4 TCR Tg mice (H-2^d^) were a generous gift from Dr. R.W. Dutton (Trudeau Institute, Saranac Lake, NY). Thy-1.2 TS1 TCR Tg mice (I-A/E^d^) were kindly provided by Dr. A. Caton (Wistar Institute, PA). Mice deficient in chemokine receptor CCR7 (CCR7^−/−^) have been described [5] and kindly provided by Dr. C. Rose (University of Virginia, VA). These mice were bred and housed in a pathogen-free environment and used at 9–13 wks of age for all experiments. To delete CD11c^+^ cells, DTR Tg mice were injected i.n. with DTx (30 ng/60 µl PBS, Sigma). All animal experiments were performed in accordance with protocols approved by the University of Virginia Animal Care and Use Committee.

### Infection of mice

Type A influenza virus, A/PR/8/34 (H1N1), was grown in day 10 chicken embryo allantoic cavities as described previously [8]. Mice were infected either i.n. (in 50 µl) with 350 egg infectious doses (EID) or intravenously (in 500 µl) via tail vein with 1.7×10^7^ EID. Inactivation of infectious influenza virus was prepared by UV-crosslinking as described previously [55]. After UV exposure of the virus suspension is complete, the samples are serially diluted 10-fold and determined for infectivity using plaque assay in MDCK cells. The non-infectious virus is delivered i.n. at 1.7 ng/ml in 50 µl.

### Intranasal instillation of FITC-ovalbumin

Mice were given i.n. either FITC-conjugated ovalbumin (FITC-Ova, 1 mg in100 µl, Moleular Probes, Eugene, OR) or unlabeled Ova (1 mg in100 µl, Sigma) as a control. The amounts of FITC-Ova taken by lung cells in uninfected mice were assessed at 1 hr post-instillation by flow cytometry and compared to unlabeled Ova. In infected mice, FITC-Ova or Ova proteins were i.n. instilled at d2 p.i. FITC^+^ cells in the lung-draining MLN or spleens were examined at 24 hr later.

### Preparation of tissue leukocytes

Preparation of lung single cell suspensions after enzymatic digestion were described elsewhere [27]. Briefly, Mice were sacrificed and lungs were perfused via the right ventricle of the heart with 5 ml sterile PBS to remove the intravascular pool of cells from the lung vasculature. The tissues were minced and then digested in 183 U/ml Type II collagenase (Worthington, Lakewood, NJ) in IMDM (Invitrogen, Gaithersburg, MD) at 37°C for 30–40 minutes. Afterwards, lung tissues were passed though a wire screen. Single cell suspensions from LN and spleens were prepared by mashing through 70 µM Cell Strainer (BD) without enzymatic digestion. Red Blood Cells (RBC) in the cell suspensions were lysed using ammonium chloride. Cells were washed and resuspended in FACS buffer containing 1× PBS, 2% FBS, 10 mM EDTA and 0.01% NaN_3_ for Ab staining or MACS buffer containing 1× PBS, 2% FBS and 10 mM EDTA for sorting. Viable nucleated cells in the suspensions were counted using a hemacytometer after exclusion of dead cells using Trypan blue dye.

### T cell and DC isolation

For CD8^+^ or CD4^+^ T cell isolation, cell suspensions from spleens were isolated using a CD8 or CD4 T cell isolation kit, according to the manufacturer's protocol (Miltenyi Biotec). For DC isolation from LN (n = 30–35 mice/sorting), CD11c^+^ cells were enriched by negative selection after incubation with a mix of specific mAbs (FTIC-conjugated mAbs against CD19, DX5 and TCRβ), and anti-FITC magnetic microbeads using MACS cell sorter (Miltenyi Biotec) in accordance with the manufacturer's instruction. The enriched populations were stained with a cocktail of mAbs specific to I-A^d^, CD103, CD11c, B220, and CD11b and sorted for CD11c^+^B220^+^MHC II^low^ (pDC), CD11c^+^B220^−^CD11b^hi^MHC II^int^ (Gr-1^+^ MoDC), CD11^+^B220^−^MHC II^hi^CD11b^+^CD103^−^ (CD11b^hi^ DC) or CD11c^+^B220^−^MHC II^hi^CD103^+^ (CD103^+^ DC) (see Fig. S9) by flow cytometry using a FACSVantage SE Turbo sorter at the Flow Cytometry Core Facility (University of Virginia).

### *In vitro* DC:T cell co-cultures

The culture medium used for DC:T cell cultures was RPMI 1640 supplemented with 10% heat-inactivated FCS, 1% Sodium Pyruvate, 2 mM L-glucose, 20 mM HEPES, 5 mM β-mercaptoenthanol and 100 µg/ml Gentamycin (all from Invitrogen). For DC:T cell cultures, 1×10^4^ DCs and 1×10^5^ T cells were cultured in a volume of 200 µl in round-bottomed 96-well plates (Corning) and incubated at 37°C for 4 days in RPMI 1640 media. Peptide-coated DC were prepared by incubating isolated DC with 1 µM peptide (HA_533–541_) for 1 hr at 37°C, followed by 2× washes to remove any free peptides prior to co-culture with T cells.

### Adoptive transfer

Purified splenic naive CL-4 Tg CD8^+^ T cells were labeled with carboxyfluorescein diacetate succinimidyl ester (CFDA-SE; Molecular Probes, Eugene, OR) as previously described[8]. A total of 1–5×10^6^ CFSE-labeled CD8^+^ T cells were i.v. injected into the tail vein of Thy-mismatched congenic mice. Recipient mice were infected with influenza virus 24 hr later.

### Antibodies

The following monoclonal antibodies (mAb) were purchased from BD-Biosciences (BD) (San Diego, CA) or eBiosciences (eBio) (San Diego, CA) (unless stated), as conjugated to FITC, PE, PE-Cy7, PerCP-Cy5.5, APC, APC-Alexa740 or Biotin: MHC class II (I-A^d^, AMS-32-1), MHC class I (H-2K^d^, SF1-1.1), Gr-1 (RB6-8C5), TCRβ (H57-597), CD1d (1B1), CD4 (L3T4), CD8α (53-6.7), CD11a (2D7), CD11b (M1/70), CD11c (HL3), CD19 (1D3), CD40 (3/23), CD43 (S7), CD43 (1B11), CD45 (30-F11), B220 or CD45R (RA3-6B2), CD80 (16-10A1), CD83 (Michel 17), CD86 (GL-1), CD115 (AFS98), DX5 (HMα2), B7-H1 (MIH5), B7-DC (TY25), β7 integrin (M293), ICOSL or B7-H2 (HK5.3), Granzyme B (GB12, Caltag), IFN-γ (XMG1.2), TNF-α (MP6-XT22), IL-4 (BVD4-1D11), IL-10 (JES5-16E3),Ki-67 (MOPC-21), CCR7 (EBI-1), SiglecF (E50-2440), F4/80 (Cl:A3, Caltag), CD103-Alexa-Fluor-647 (2E7, Biolegend, San Diego, CA), mPDCA (Miltenyi Biotec, Auburn, CA), isotype control antibodies. Anti-mouse CD16/32 used for Fc receptor blocking was isolated and purified in our laboratory. For biotinylated mAbs, samples were incubated with streptavidin (SA)-RPE, SA-APC or SA-APC-Cy7.

### Flow cytometry analysis and intracellular staining

Cell suspensions were blocked with 2.4G2 and then incubated with specific mAbs or isotype controls for 25 min on ice. For surface marker staining, cells were fixed in FACSLysing Solution (BD). For intracellular cytokine staining, cells obtained from *in vitro* cultures were incubated for 4–5 hr with synthetic peptide (HA_533–541_) for CD8^+^ T cells with Thy-mismatched splenocytes as an additional APC or with 50 ng/ml PMA and 750 ng/ml Ionomycin (both from Sigma) for CD4^+^ T cells in the presence of monensin (BD) in a tissue culture incubator at 37°C. After surface staining for 25 min with the corresponding cocktail of fluorescently labeled antibodies, cells were resuspended in Cytofix/Cytoperm solution (BD), and intracellular cytokine staining was performed according to the manufacturer's protocol. Granzyme B was detected directly after surface staining. For the detection of nucleocapsid (NP) viral protein, mouse monoclonal anti-NP antibody (clone H16, a gift from Dr. Walter Gerhard, Wistar institute, PA) was conjugated to Alexa-555 (Molecular Probe, Eugene, OR) according to the manufacturer's instruction and used to intracellularly stain for NP. For Ki-67 staining, surface-stained cells initially were fixed in 1× FACSLysing solution, followed by permeabilization using 1× Perm II solution (BD) for 15 min at room temperature (RT). The fixed/permeabilized cells were incubated with PE-labeled anti-Ki-67 or isotype-matched mAbs (BD) for an additional 30 min at RT. Flow cytometry was performed on either FACSCalibur or FACSCanto flow cytometers (BD, Mountain View, CA), and data were analyzed using Flowjo (Tree star).

### BrdU labeling and detection

Mice were given 1 mg/250 µl BrdU by i.p. injection on the day of influenza virus infection and continuously fed in drinking water (0.4 mg/ml) with daily changes. Tissue cells were prepared for cell surface antibody Ab labeling and BrdU staining was performed using the BD FastImmune BrdU kit according to the manufacturer's instructions (BD).

### Statistics

A two-tailed unpaired Student *t* test was used to analyze differences in mean values between groups. These statistical analyses were performed using the GraphPad Prism3 Software Program for Macintosh. All results are expressed as means±SD. Values of P<0.05 were considered statistically significant.

## Supporting Information

Figure S1 Characterization of CD11c+ cells in the normal lung. (A) Flow cytometry-based categorization of RDC subpopulations. Hematopoietic origin cells in the lung suspensions (panel a) are identified as CD45+ (panel b). Among the CD11c+ cells (panel c), the highly auto-fluorescent alveolar macrophages (AM) [SSChiMHC II− (panel d)] and B220+ plasmacytoid DC (pDC - mPDCA-1+Gr-1+, panel e) are excluded from further analyses. The remaining lung leukocytes are further divided into three subsets; CD103+ RDC (panel f), CD103-CD11bhiMHC II+/− (MoRDC, panel g) and CD103-CD11bhiMHC IIhi (CD11bhi RDC, panel h). (B) Identification of AM in the lung. Single cell suspensions from normal lung are stained with a cocktail of mAbs specific to CD45, CD11c, SiglecF and MHC II. CD45+CD11c+ cells are examined for SigecF expression (top panel) and SSC property (bottom panel) along with MHC II expression. (C) Diagrams depicting the cellular composition of the CD11c+ cells in the single cell lung suspensions (mean±SD; n = 5). (D) Surface marker expression on subsets of RDC. Lung-residing DC subsets – CD103+ RDC (top), CD11bhi RDC (middle), and MoRDC (bottom) – are examined at d0 (open) or d2 p.i. (gray) for surface expression of various markers tested. Vertical lines (□) indicate the median values of isotype-matched control antibodies (black) for surface marker staining.
(18.84 MB TIF)

Figure S2 Strategy for identifying DC subsets in the MLN draining lung. (A–C) Gating strategy for identifying DC subsets in the MLN of uninfected or influenza-infected mice. Total leukocytes isolated from the MLN of uninfected (column A) or i.n. infected wild type (wt, column B) or B cell-deficient (µMT, column C) mice at d3 p.i. are stained with a cocktail of mAbs specific to TCRβ, CD11c, CD11b, B220, CD103 and MHC II. T cells are first gated out to discriminate CD11c+ cells (row i). Subsequently, TCRβ-CD11c+ cells (row ii) are further analyzed for B220 vs MHC II expression, which results in the identification of pDC (B220+MHC IIloGr−1+CD19−) as well as B cells (B220+MHC IIhiGr-1-CD19+) (row iii). TCRβ-CD11c+B220− cells (row iii) are further examined for CD11b vs MHC II expression, dividing them into two groups: MHC II−/lo and MHC IIhi (row iv). Cells within MHC II−/lo fraction can be classified into Gr-1+ MoDC (group III) and Gr-1− MoDC (row v). Lastly, cells expressing high levels of MHC II are further divided into two subgroups based on CD103 and CD11b expression; CD11bhiCD103− cells identified as ‘CD11bhi DC’ (group II) and CD11b+/−CD103+ cells as ‘CD103+ DC’ (group I).
(11.48 MB TIF)

Figure S3 Identification and phenotypic characteristics of LN-resident CD8αα+ DC. Total LN cells were liberated, after enzymatic digestion, from the MLN of infected mice at indicated days post infection with influenza virus as previously described [2], [7]. The liberated single cell suspensions were stained with a cocktail of mAbs specific to CD11c, CD11b, CD103, CD8α and MHC II (A) or accessory surface markers (B). (A) CD11chiMHC IIint (R1) were examined for the expression of CD8α and CD11b. The CD8αhi population in R1 gates, representing the authentic LN-resident CD8αα+ DC subset, are identified in the node of uninfected and infected animals. CD11cmed-hi MHC IIhi (R2) are also examined for their expression of either CD8α and CD11b or CD103 and CD11b. Of note, CD8α expressing CD103+ and CD11bhi DC subsets in R2 gates gradually increased as infection progressed (compare 3 dpi to 0 dpi). (B) The CD8αα+ LNDC express higher levels of CD8α and CD11c and lower levels of CD80, CD83, CD86, B7-H1, B7-DC and MHC II than those of CD103+ or CD11bhi DC in the MLN of infected mice when analyzed at d3 p.i.
(5.31 MB TIF)

Figure S4 Morphologic and phenotypic characteristics of DC subsets in the MLN of infected mice. DC subsets accumulating in the MLN of influenza-infected mice at d3 p.i. are identified as described in the Figure S2 and examined for their size (FSC), intracellular complexity (SSC), and surface maker expression after staining with the indicated mAbs.
(2.76 MB TIF)

Figure S5 Active viral nucleoprotein synthesis in infected CD103+ RDC in vitro and in vivo. (A) CD103+ RDC were single-cell sorted from uninfected lungs and incubated with A/PR/8 for 1 hr in vitro. After additional 18 hrs of incubation, the CD103+ RDC were fixed and examined for intracellular expression of MHC II (green, left) and viral NP (red, right) under confocal microscope. (B) A/PR/8-infected mice were given i.n. FITC-Ova at d2 p.i., and MLN were excised at d3 p.i. and frozen for immunofluorecence study. Migrant CD103+ RDC (NP+ and FITC-Ova+) in the MLN were identified by Flow cytometry (See Figure 3 A and B) and examined for intracellular expression of NP (red) under confocal microscope.
(12.15 MB TIF)

Figure S6 Accumulation of CD11bhi DC and CD103+ DC in the MLN of influenza-infected mice is as a result of the emigration of the corresponding counterparts in the lung, and is dependent on CCR7. (A) Cell division history of DC subsets in the MLN of infected mice. Mice were i.n. infected with influenza virus and given BrdU in drinking water while during the 3 d infection period (controls without BrdU in gray). A representative analysis of % BrdU+ DC subsets in MLN is shown (n = 4). (B) Lung-residing CD11bhi and CD103+ RDC are necessary for the accumulation of the corresponding DC subsets in the MLN of influenza-infected mice. DTR mice are i.n. administered with either DTx or PBS one day prior to i.n. influenza virus infection. At d3 p.i., DC subsets in the MLN of infected mice are identified (see Fig. 4E), and the absolute numbers of DC subsets and total cellularity in the MLN of infected DTR mice are depicted (mean±SD; n = 4–8 mice/group). (C and D) CCR7-deficient mice failed to show the accumulation of CD11bhi DC and CD103+ DC, but not Gr-1+ MoDC, in the MLN of influenza-infected mice. CCR7+/+ or CCR7−/− mice i.n. infected 3 d earlier with influenza virus are examined for the accumulated DC subsets in the MLN of infected mice. Identification (C) and the numbers of DC subsets and total cellularity (D) in the MLN of infected mice are depicted (mean±SD; n = 3–5 mice).
(12.90 MB TIF)

Figure S7 Tempo of the antigen-specific naïve CD8+ T cell proliferation in the MLN of infected mice. Mice (Thy1.1) received CFSE-labeled Thy1.2 CL-4 CD8+ T cells were i.n. infected with influenza virus and examined for the proliferation profiles (dilution of CFSE contents) of naïve CL-4 CD8+ T cells in the MLN at indicated days p.i. Data shown are gated on Thy1.2+ CD8+ T cells.
(0.36 MB TIF)

Figure S8 CCR7-deficient mice are impaired in supporting the proliferative expansion of CCR7-competent naïve CL-4 tg CD8+ T cells. (A) CCR7−/− mice (Thy1.2) received CFSE-labeled Thy1.1 CCR7+/+ CL-4 CD8+ T cells were i.n. infected 4 days earlier, and the magnitude of expansion and dilution of CFSE contents in naive CL-4 CD8+ T cells in the MLN are examined. Data shown are gated on Thy1.1+ CD8+ T cells, and a representative of 3 independent experiments is depicted. (B) CCR7 deficiency on host LNDC exhibits a comparable capability to activate CCR7-competent donor naive CD8 T cells compared to CCR7+/+ LNDC when influenza virus were delivered via intravenous route. WT or CCR7−/− mice were received CFSE-labeled Thy-mismatched CCR7+/+ A/PR/8 HA-specific TCR tg naive CD8 T cells and infected with A/PR/8 via intravenous route 24 hr later. The division profiles and proliferative expansion of the HA-specific CD8 T cells in the MLNs and spleens were examined at d3 p.i.
(3.05 MB TIF)

Figure S9 A 4-way cell sorting of DC subsets in the MLN of infected mice. (A–D) Single cells are prepared from the MLN of i.n. infected mice 3 days earlier with influenza virus. To enrich CD11c+ cells from the total leukocytes in the MLN, T, B and NK cells are depleted using a cocktail of magnetic beads directed at TCRβ, CD19 or DX5 as described in Materials and Methods. The resulting fraction containing the enriched CD11c+ cells is surface-stained with a cocktail of mAbs specific for CD11c, B220, CD11b, CD103, or MHC II, and subjected to a FACS-based 4-way cell sorting. DCs are identified by CD11c expression. The CD11c+ cells are initially displayed for B220 vs MHC II for identifying B220+ pDC (A). The B220-CD11c+ cells are then displayed for CD11b vs MHC II, which results in two distinct populations based on the level of MHC II expression; CD11bhiMHC IIlo vs CD11blo-hiMHC IIhi cells. The former is mainly composed of Gr-1+ DC (see Fig. S2) and sorted as Gr-1+ MoDC (B). Afterwards, the MHC IIhi cells are further divided into two groups based on CD103 expression; CD103− DC (C) vs CD103+ DC (D). MHC IIhiCD103− cells are also largely CD11bhi cells and sorted as CD11bhi DC (C). Purity of the sorted cells is determined by flow cytometry using the respective subsets of post-sorted DC, which results in greater than 97% of homogeneity in all experiments (right panels).
(5.38 MB TIF)

Figure S10 Proliferation of CL-4 CD8+ T cells stimulated by peptide-pulsed either pDC or Gr-1+ MoDC. (A and B) B220+ pDC (A) or Gr-1+ MoDC (B) are sorted from the MLN of infected mice 3 days earlier with infectious influenza virus and pulsed with HA533–541 peptide (open) or left untreated (filled) for 1 hr. pDC or Gr-1+ MoDC are then co-cultured with CFSE-labeled naïve CL-4 CD8+ T cells in culture. At day 4 the dilution of CFSE contents in CL-4 CD8+ T cells is examined by flow cytometry. A representative of 3 independent experiments is depicted.
(1.34 MB TIF)

Figure S11 Effector cytokine production of CD4+ T cells stimulated by either CD11bhi or CD103+ DC subsets. (A–C) CD11bhi DC (top panel) and CD103+ DC (lower panel) in the MLN of i.n. infected mice 3 days earlier with infectious influenza virus are sorted and co-cultured with CFSE-labeled HA-specific naïve TCR tg TS1 CD4+ T cells in culture. Four days later, CD4+ T cells are restimulated with PMA/ionomycin for an additional 4 hr in the presence of monensin and intracellularly stained for IFN-γ (A), IL-4 (B) or IL-10 (C) secretion. A representative of 3 independent experiments is depicted.
(2.01 MB TIF)

Figure S12 CD8αα+ LNDC are less efficient than migrant CD103+ and CD11bhi RDC at stimulating naïve virus specific CD8+ T cells. (A) The indicated DC subsets were isolated, after enzymatic treatments, from the MLN of mice infected 3 d earlier with influenza virus (A/PR/8) [i.e., Gr-1+ MoDC (i), LN-resident CD8αα+ DC (ii), CD11bhi DC (iii) and CD103+ DC (iv)]. The DC subsets were co-cultured with CFSE-labeled HA533–541 (A/PR/8/34)-specific naïve TCR tg CD8+ T cells (Cl-4) for 4 days. Proliferation profile and total recovery (see inserts) of viable CD8+ T cells stimulated by the MLN-derived DC subsets are depicted. (B) As a control in companion experiments, the DC subsets used in (A) were pulsed with HA210–219 (A/JAPAN/57) and were subsequently co-cultured for 4 days with CFSE-labeled HA 210–219-specific naïve TCR tg CD8+ T cells (Demi-4). These CD8+ TCR tg T cells do not cross-react with the A/PR/8/34 hemagglutinin.
(7.09 MB TIF)
