# Supplementary figures and images for: Respiratory Dendritic Cell Subsets Differ in Their Capacity to Support the Induction of Virus-Specific Cytotoxic CD8+ T Cell Responses
Source: PLoS One. 2009 Jan 15;4(1):e4204. doi: 10.1371/journal.pone.0004204 (PMC2615220; doi:10.1371/journal.pone.0004204)

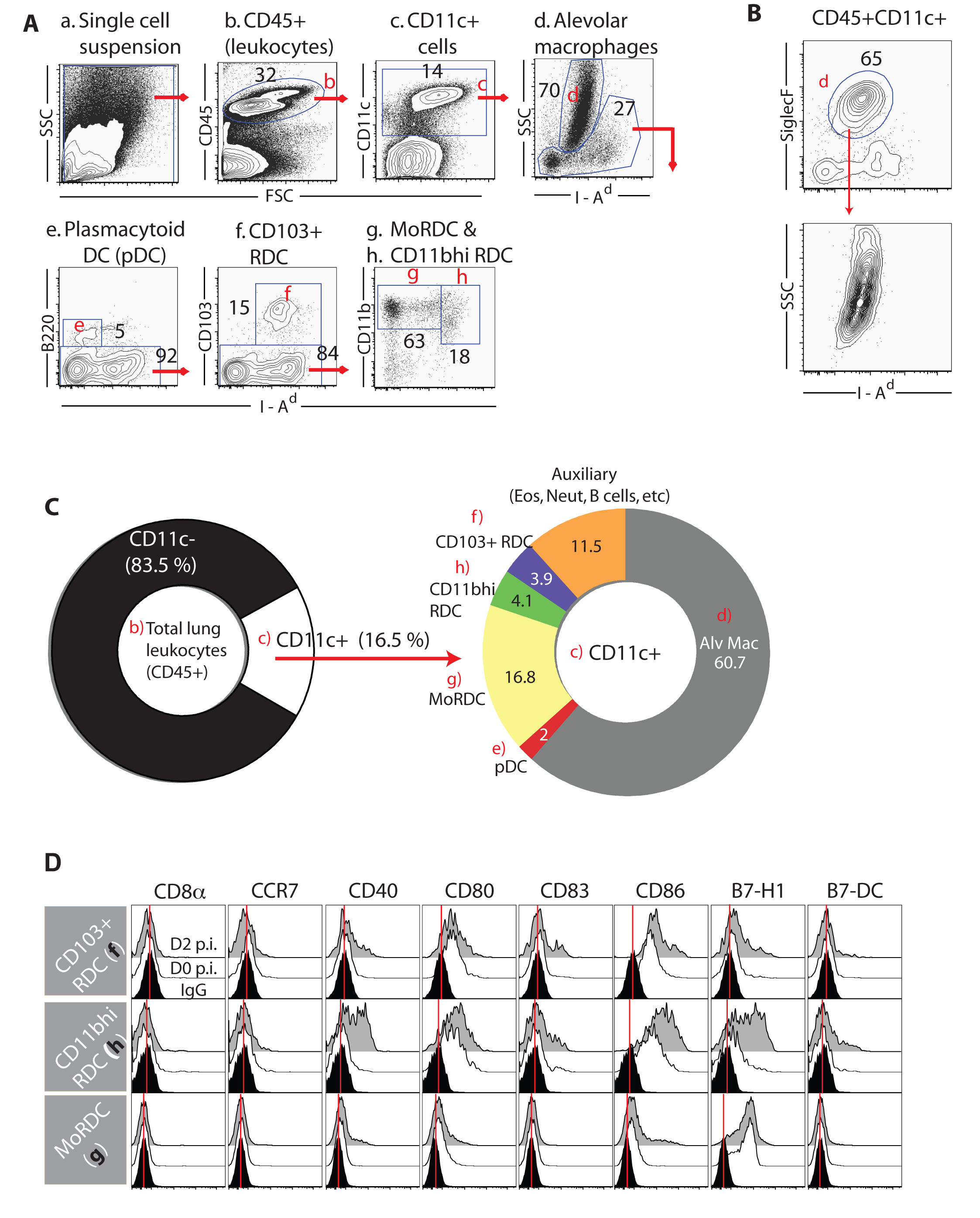

Supplement: Figure S1 — Characterization of CD11c+ cells in the normal lung. (A) Flow cytometry-based categorization of RDC subpopulations. Hematopoietic origin cells in the lung suspensions (panel a) are identified as CD45+ (panel b). Among the CD11c+ cells (panel c), the highly auto-fluorescent alveolar macrophages (AM) [SSChiMHC II− (panel d)] and B220+ plasmacytoid DC (pDC - mPDCA-1+Gr-1+, panel e) are excluded from further analyses. The remaining lung leukocytes are further divided into three subsets; CD103+ RDC (panel f), CD103-CD11bhiMHC II+/− (MoRDC, panel g) and CD103-CD11bhiMHC IIhi (CD11bhi RDC, panel h). (B) Identification of AM in the lung. Single cell suspensions from normal lung are stained with a cocktail of mAbs specific to CD45, CD11c, SiglecF and MHC II. CD45+CD11c+ cells are examined for SigecF expression (top panel) and SSC property (bottom panel) along with MHC II expression. (C) Diagrams depicting the cellular composition of the CD11c+ cells in the single cell lung suspensions (mean±SD; n = 5). (D) Surface marker expression on subsets of RDC. Lung-residing DC subsets – CD103+ RDC (top), CD11bhi RDC (middle), and MoRDC (bottom) – are examined at d0 (open) or d2 p.i. (gray) for surface expression of various markers tested. Vertical lines (□) indicate the median values of isotype-matched control antibodies (black) for surface marker staining. (18.84 MB TIF) [file pone.0004204.s001.tif]

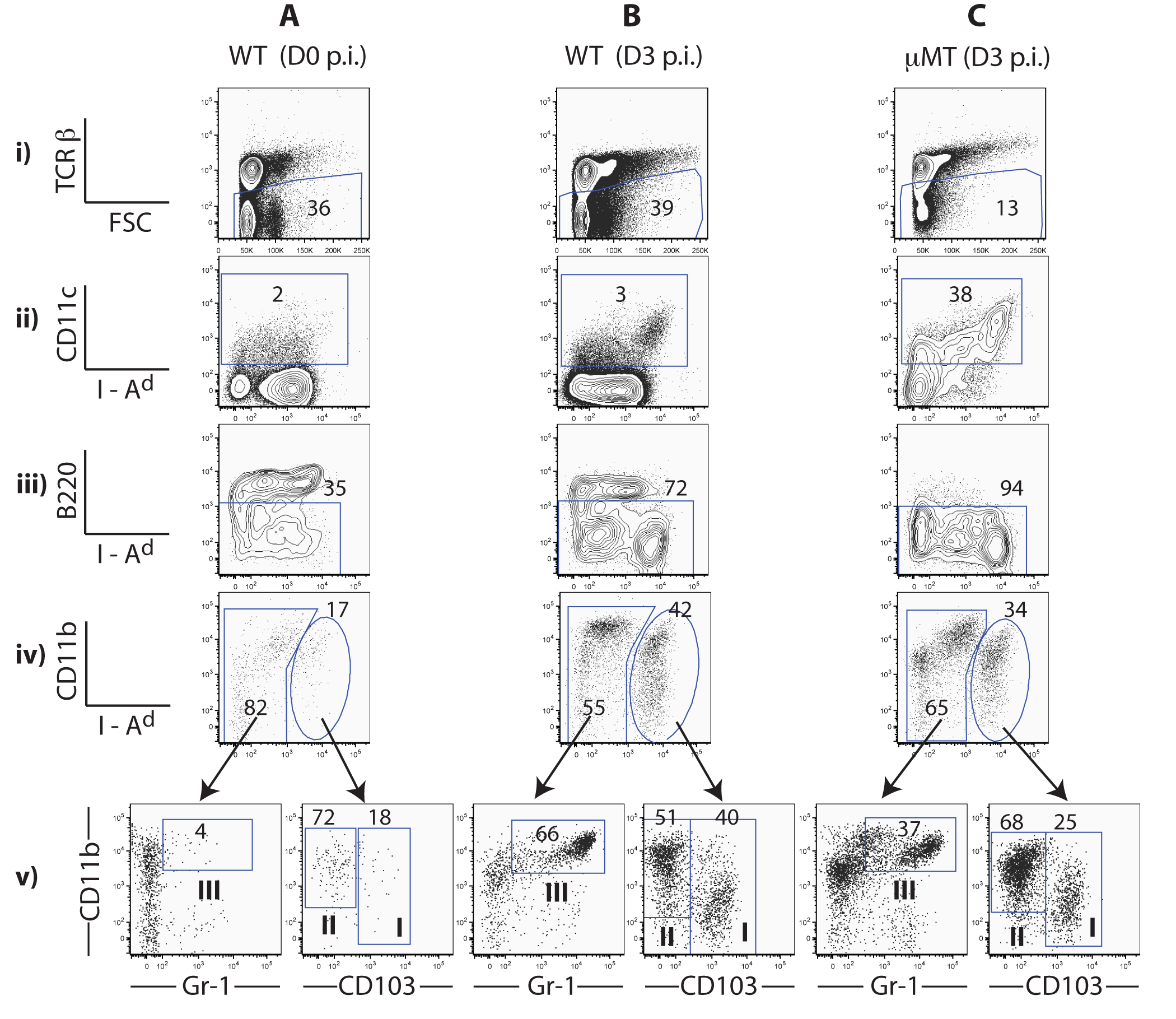

Supplement: Figure S2 — Strategy for identifying DC subsets in the MLN draining lung. (A–C) Gating strategy for identifying DC subsets in the MLN of uninfected or influenza-infected mice. Total leukocytes isolated from the MLN of uninfected (column A) or i.n. infected wild type (wt, column B) or B cell-deficient (µMT, column C) mice at d3 p.i. are stained with a cocktail of mAbs specific to TCRβ, CD11c, CD11b, B220, CD103 and MHC II. T cells are first gated out to discriminate CD11c+ cells (row i). Subsequently, TCRβ-CD11c+ cells (row ii) are further analyzed for B220 vs MHC II expression, which results in the identification of pDC (B220+MHC IIloGr−1+CD19−) as well as B cells (B220+MHC IIhiGr-1-CD19+) (row iii). TCRβ-CD11c+B220− cells (row iii) are further examined for CD11b vs MHC II expression, dividing them into two groups: MHC II−/lo and MHC IIhi (row iv). Cells within MHC II−/lo fraction can be classified into Gr-1+ MoDC (group III) and Gr-1− MoDC (row v). Lastly, cells expressing high levels of MHC II are further divided into two subgroups based on CD103 and CD11b expression; CD11bhiCD103− cells identified as ‘CD11bhi DC’ (group II) and CD11b+/−CD103+ cells as ‘CD103+ DC’ (group I). (11.48 MB TIF) [file pone.0004204.s002.tif]

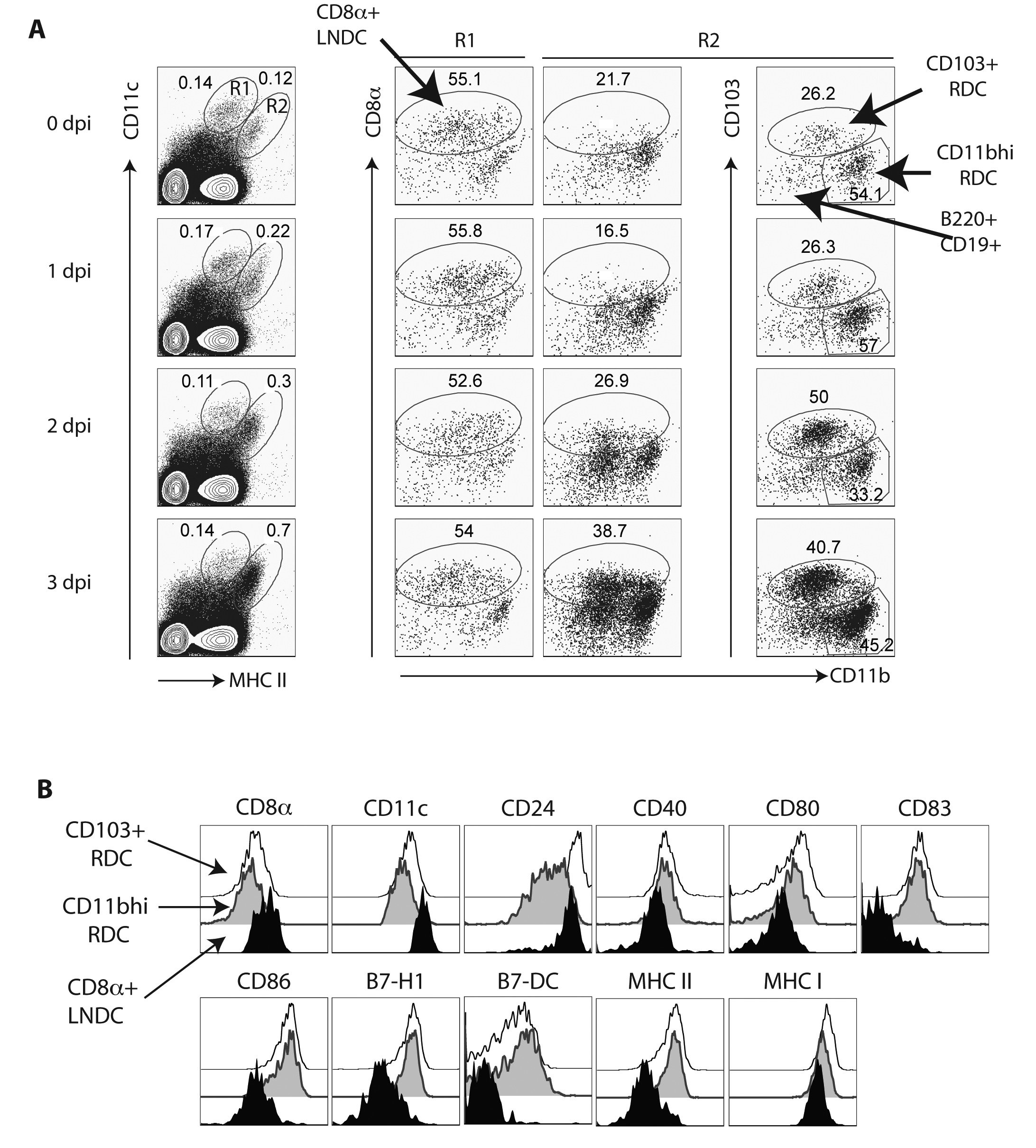

Supplement: Figure S3 — Identification and phenotypic characteristics of LN-resident CD8αα+ DC. Total LN cells were liberated, after enzymatic digestion, from the MLN of infected mice at indicated days post infection with influenza virus as previously described [2], [7]. The liberated single cell suspensions were stained with a cocktail of mAbs specific to CD11c, CD11b, CD103, CD8α and MHC II (A) or accessory surface markers (B). (A) CD11chiMHC IIint (R1) were examined for the expression of CD8α and CD11b. The CD8αhi population in R1 gates, representing the authentic LN-resident CD8αα+ DC subset, are identified in the node of uninfected and infected animals. CD11cmed-hi MHC IIhi (R2) are also examined for their expression of either CD8α and CD11b or CD103 and CD11b. Of note, CD8α expressing CD103+ and CD11bhi DC subsets in R2 gates gradually increased as infection progressed (compare 3 dpi to 0 dpi). (B) The CD8αα+ LNDC express higher levels of CD8α and CD11c and lower levels of CD80, CD83, CD86, B7-H1, B7-DC and MHC II than those of CD103+ or CD11bhi DC in the MLN of infected mice when analyzed at d3 p.i. (5.31 MB TIF) [file pone.0004204.s003.tif]

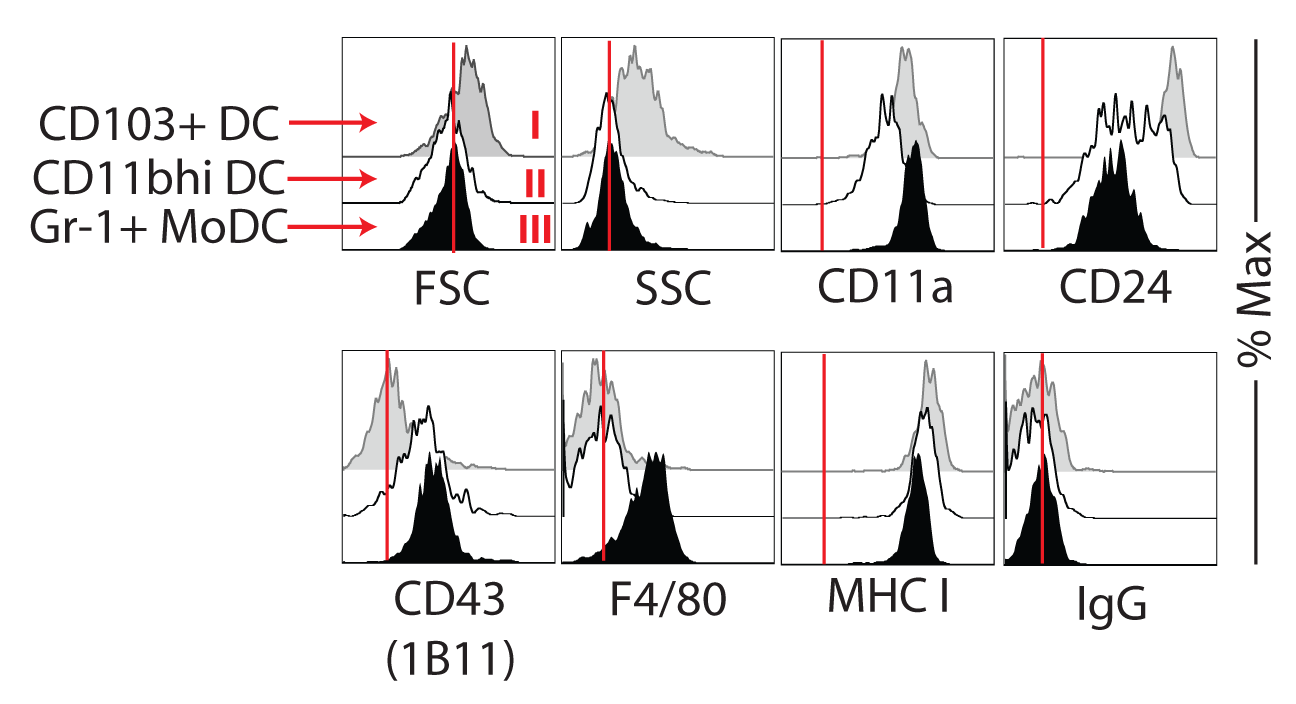

Supplement: Figure S4 — Morphologic and phenotypic characteristics of DC subsets in the MLN of infected mice. DC subsets accumulating in the MLN of influenza-infected mice at d3 p.i. are identified as described in the Figure S2 and examined for their size (FSC), intracellular complexity (SSC), and surface maker expression after staining with the indicated mAbs. (2.76 MB TIF) [file pone.0004204.s004.tif]

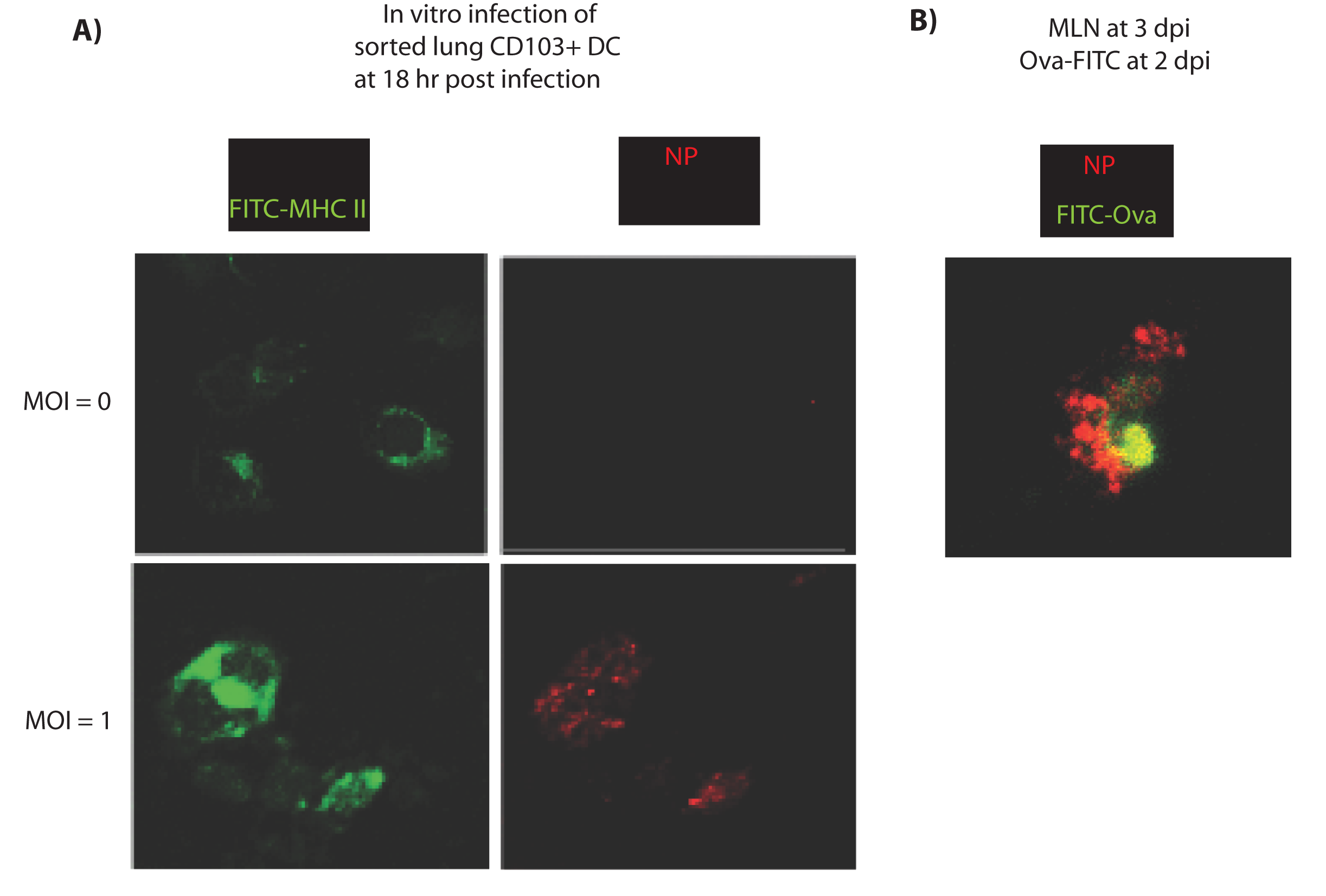

Supplement: Figure S5 — Active viral nucleoprotein synthesis in infected CD103+ RDC in vitro and in vivo. (A) CD103+ RDC were single-cell sorted from uninfected lungs and incubated with A/PR/8 for 1 hr in vitro. After additional 18 hrs of incubation, the CD103+ RDC were fixed and examined for intracellular expression of MHC II (green, left) and viral NP (red, right) under confocal microscope. (B) A/PR/8-infected mice were given i.n. FITC-Ova at d2 p.i., and MLN were excised at d3 p.i. and frozen for immunofluorecence study. Migrant CD103+ RDC (NP+ and FITC-Ova+) in the MLN were identified by Flow cytometry (See Figure 3 A and B) and examined for intracellular expression of NP (red) under confocal microscope. (12.15 MB TIF) [file pone.0004204.s005.tif]

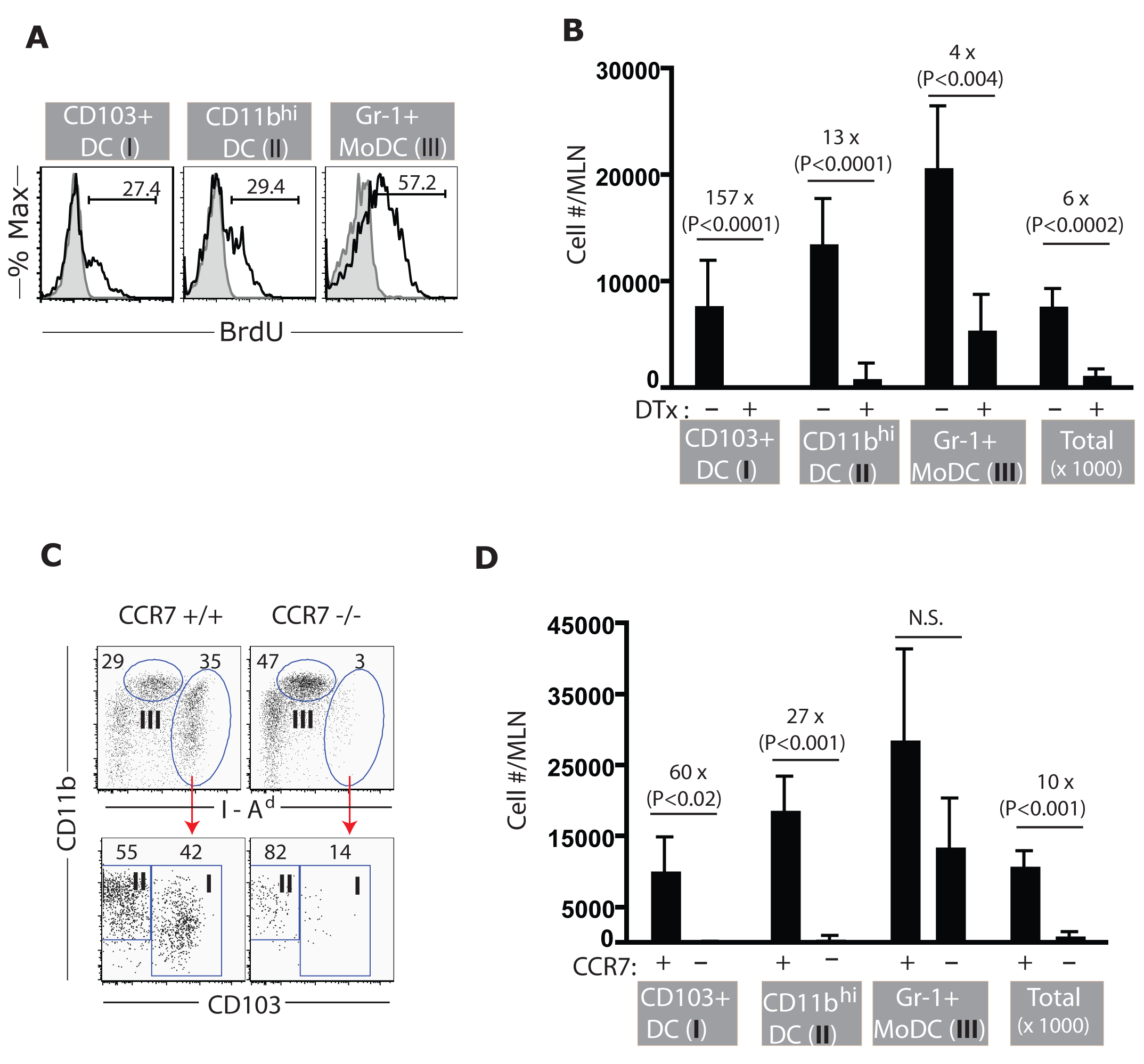

Supplement: Figure S6 — Accumulation of CD11bhi DC and CD103+ DC in the MLN of influenza-infected mice is as a result of the emigration of the corresponding counterparts in the lung, and is dependent on CCR7. (A) Cell division history of DC subsets in the MLN of infected mice. Mice were i.n. infected with influenza virus and given BrdU in drinking water while during the 3 d infection period (controls without BrdU in gray). A representative analysis of % BrdU+ DC subsets in MLN is shown (n = 4). (B) Lung-residing CD11bhi and CD103+ RDC are necessary for the accumulation of the corresponding DC subsets in the MLN of influenza-infected mice. DTR mice are i.n. administered with either DTx or PBS one day prior to i.n. influenza virus infection. At d3 p.i., DC subsets in the MLN of infected mice are identified (see Fig. 4E), and the absolute numbers of DC subsets and total cellularity in the MLN of infected DTR mice are depicted (mean±SD; n = 4–8 mice/group). (C and D) CCR7-deficient mice failed to show the accumulation of CD11bhi DC and CD103+ DC, but not Gr-1+ MoDC, in the MLN of influenza-infected mice. CCR7+/+ or CCR7−/− mice i.n. infected 3 d earlier with influenza virus are examined for the accumulated DC subsets in the MLN of infected mice. Identification (C) and the numbers of DC subsets and total cellularity (D) in the MLN of infected mice are depicted (mean±SD; n = 3–5 mice). (12.90 MB TIF) [file pone.0004204.s006.tif]

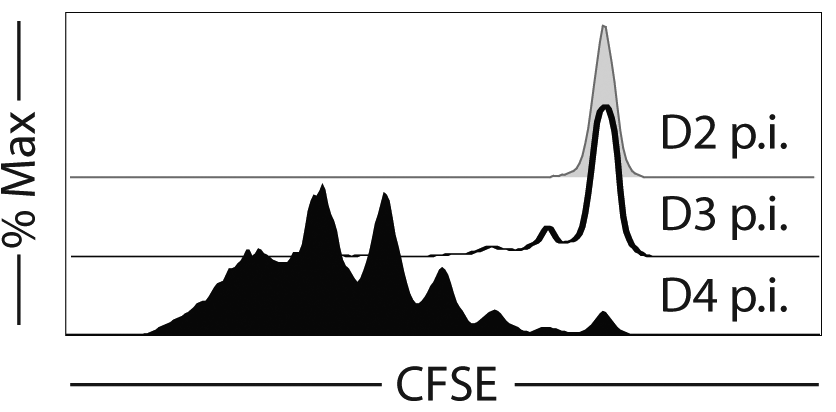

Supplement: Figure S7 — Tempo of the antigen-specific naïve CD8+ T cell proliferation in the MLN of infected mice. Mice (Thy1.1) received CFSE-labeled Thy1.2 CL-4 CD8+ T cells were i.n. infected with influenza virus and examined for the proliferation profiles (dilution of CFSE contents) of naïve CL-4 CD8+ T cells in the MLN at indicated days p.i. Data shown are gated on Thy1.2+ CD8+ T cells. (0.36 MB TIF) [file pone.0004204.s007.tif]

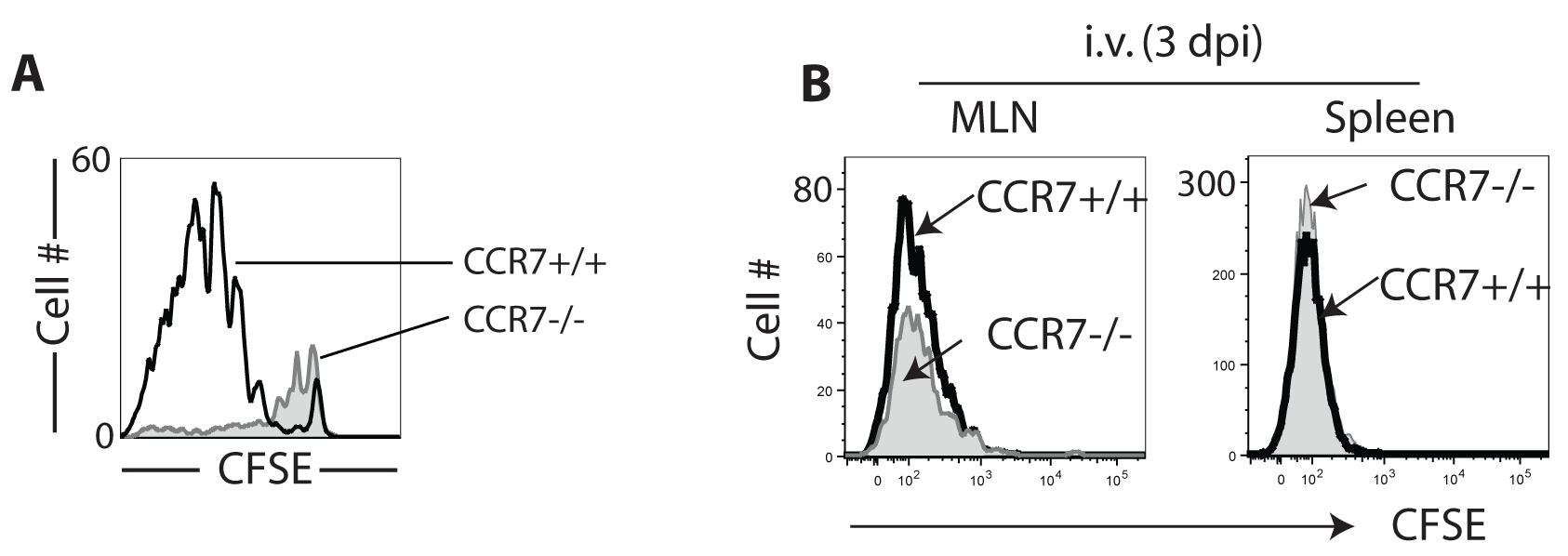

Supplement: Figure S8 — CCR7-deficient mice are impaired in supporting the proliferative expansion of CCR7-competent naïve CL-4 tg CD8+ T cells. (A) CCR7−/− mice (Thy1.2) received CFSE-labeled Thy1.1 CCR7+/+ CL-4 CD8+ T cells were i.n. infected 4 days earlier, and the magnitude of expansion and dilution of CFSE contents in naive CL-4 CD8+ T cells in the MLN are examined. Data shown are gated on Thy1.1+ CD8+ T cells, and a representative of 3 independent experiments is depicted. (B) CCR7 deficiency on host LNDC exhibits a comparable capability to activate CCR7-competent donor naive CD8 T cells compared to CCR7+/+ LNDC when influenza virus were delivered via intravenous route. WT or CCR7−/− mice were received CFSE-labeled Thy-mismatched CCR7+/+ A/PR/8 HA-specific TCR tg naive CD8 T cells and infected with A/PR/8 via intravenous route 24 hr later. The division profiles and proliferative expansion of the HA-specific CD8 T cells in the MLNs and spleens were examined at d3 p.i. (3.05 MB TIF) [file pone.0004204.s008.tif]

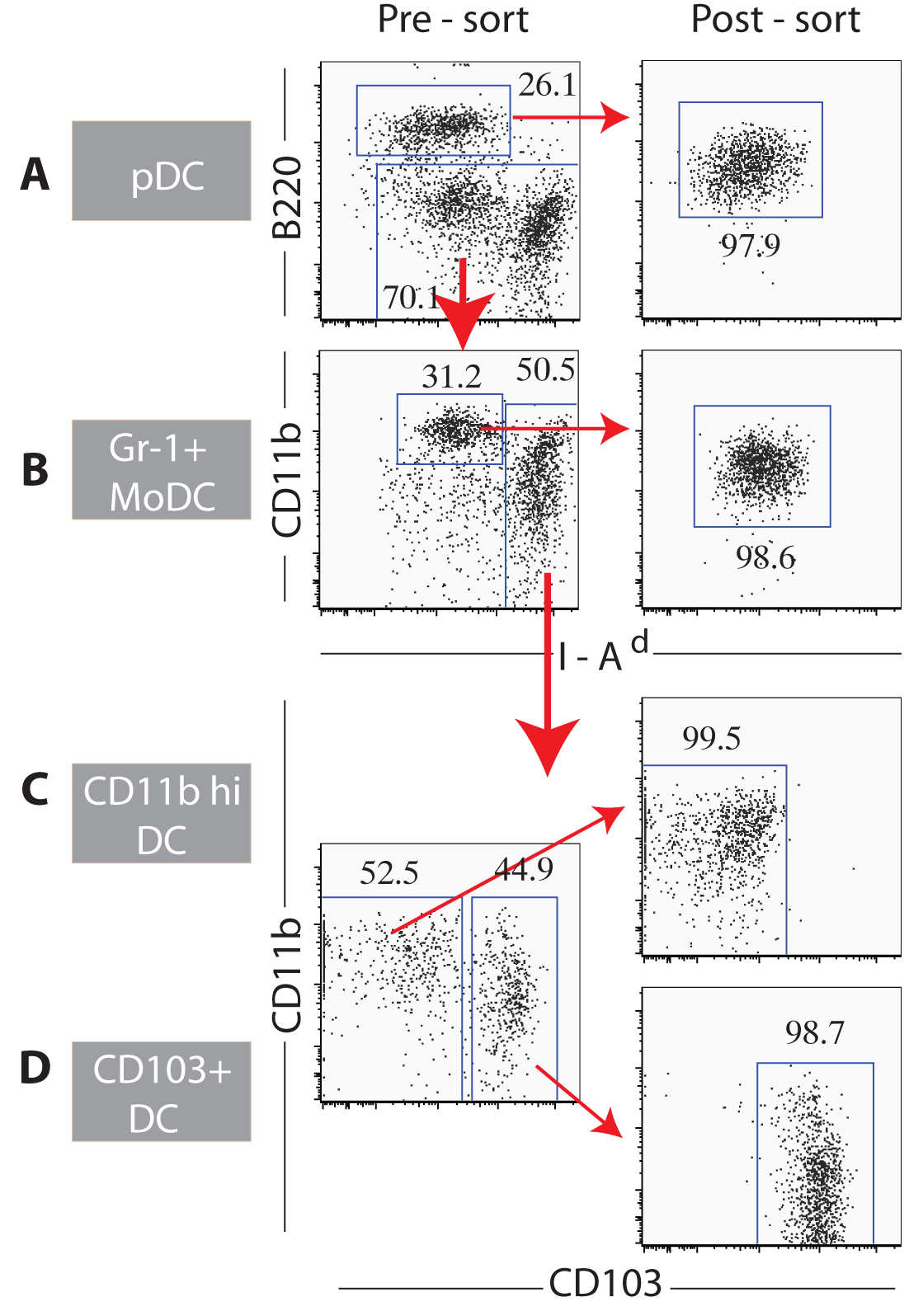

Supplement: Figure S9 — A 4-way cell sorting of DC subsets in the MLN of infected mice. (A–D) Single cells are prepared from the MLN of i.n. infected mice 3 days earlier with influenza virus. To enrich CD11c+ cells from the total leukocytes in the MLN, T, B and NK cells are depleted using a cocktail of magnetic beads directed at TCRβ, CD19 or DX5 as described in Materials and Methods. The resulting fraction containing the enriched CD11c+ cells is surface-stained with a cocktail of mAbs specific for CD11c, B220, CD11b, CD103, or MHC II, and subjected to a FACS-based 4-way cell sorting. DCs are identified by CD11c expression. The CD11c+ cells are initially displayed for B220 vs MHC II for identifying B220+ pDC (A). The B220-CD11c+ cells are then displayed for CD11b vs MHC II, which results in two distinct populations based on the level of MHC II expression; CD11bhiMHC IIlo vs CD11blo-hiMHC IIhi cells. The former is mainly composed of Gr-1+ DC (see Fig. S2) and sorted as Gr-1+ MoDC (B). Afterwards, the MHC IIhi cells are further divided into two groups based on CD103 expression; CD103− DC (C) vs CD103+ DC (D). MHC IIhiCD103− cells are also largely CD11bhi cells and sorted as CD11bhi DC (C). Purity of the sorted cells is determined by flow cytometry using the respective subsets of post-sorted DC, which results in greater than 97% of homogeneity in all experiments (right panels). (5.38 MB TIF) [file pone.0004204.s009.tif]

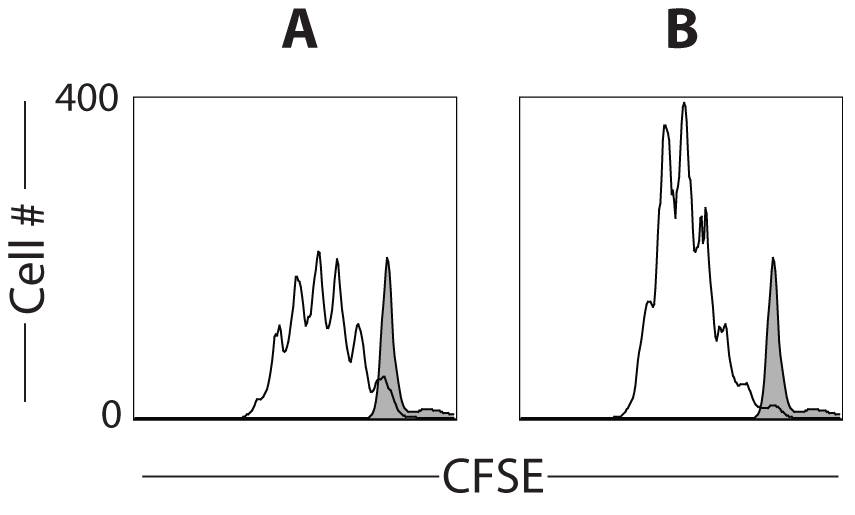

Supplement: Figure S10 — Proliferation of CL-4 CD8+ T cells stimulated by peptide-pulsed either pDC or Gr-1+ MoDC. (A and B) B220+ pDC (A) or Gr-1+ MoDC (B) are sorted from the MLN of infected mice 3 days earlier with infectious influenza virus and pulsed with HA533–541 peptide (open) or left untreated (filled) for 1 hr. pDC or Gr-1+ MoDC are then co-cultured with CFSE-labeled naïve CL-4 CD8+ T cells in culture. At day 4 the dilution of CFSE contents in CL-4 CD8+ T cells is examined by flow cytometry. A representative of 3 independent experiments is depicted. (1.34 MB TIF) [file pone.0004204.s010.tif]

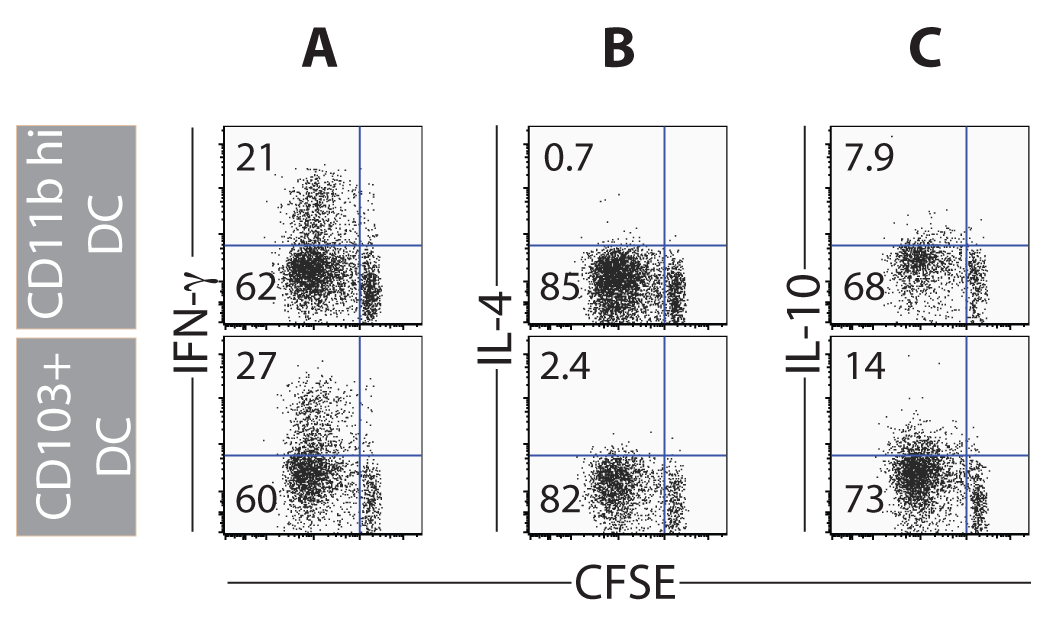

Supplement: Figure S11 — Effector cytokine production of CD4+ T cells stimulated by either CD11bhi or CD103+ DC subsets. (A–C) CD11bhi DC (top panel) and CD103+ DC (lower panel) in the MLN of i.n. infected mice 3 days earlier with infectious influenza virus are sorted and co-cultured with CFSE-labeled HA-specific naïve TCR tg TS1 CD4+ T cells in culture. Four days later, CD4+ T cells are restimulated with PMA/ionomycin for an additional 4 hr in the presence of monensin and intracellularly stained for IFN-γ (A), IL-4 (B) or IL-10 (C) secretion. A representative of 3 independent experiments is depicted. (2.01 MB TIF) [file pone.0004204.s011.tif]

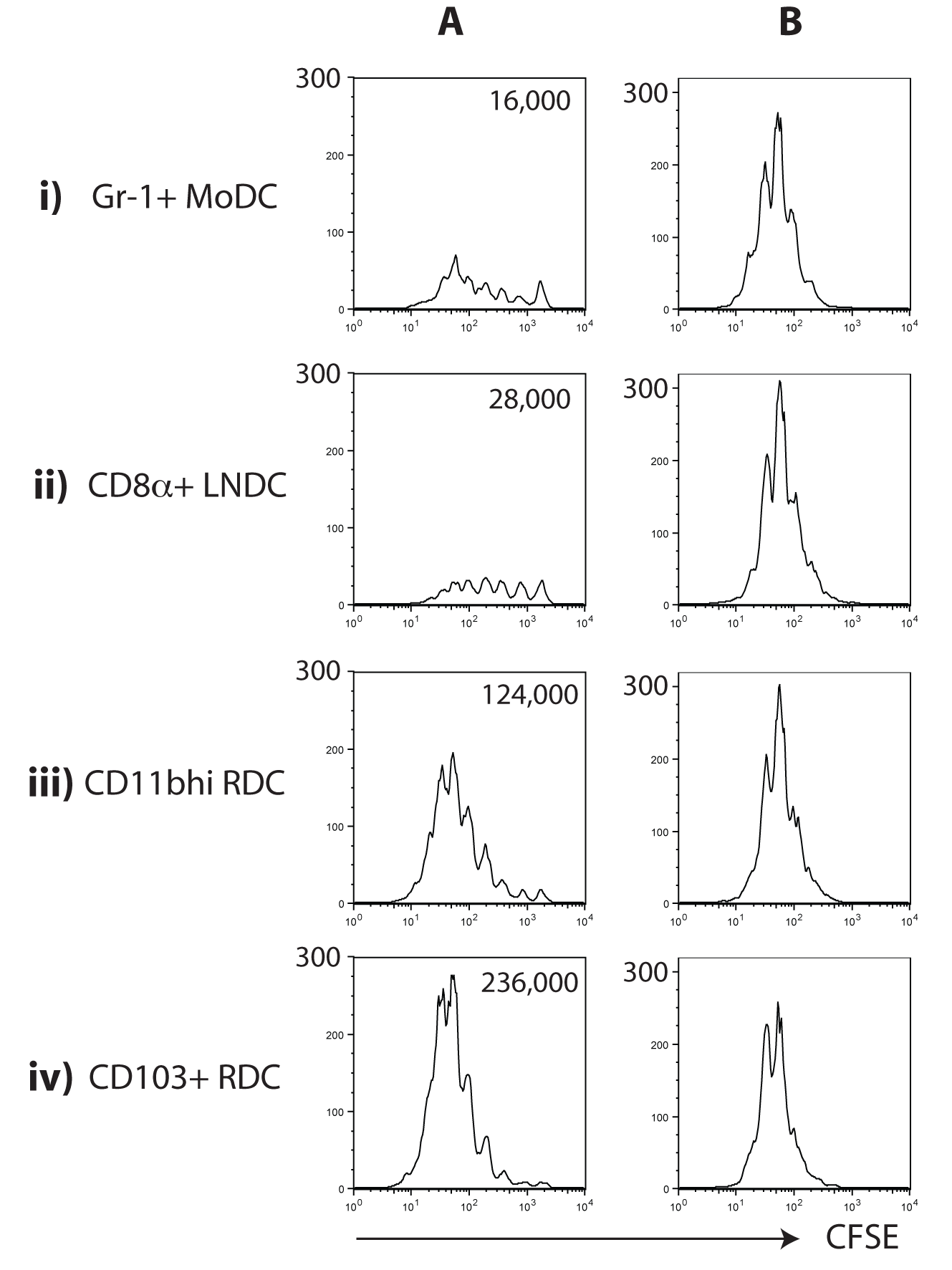

Supplement: Figure S12 — CD8αα+ LNDC are less efficient than migrant CD103+ and CD11bhi RDC at stimulating naïve virus specific CD8+ T cells. (A) The indicated DC subsets were isolated, after enzymatic treatments, from the MLN of mice infected 3 d earlier with influenza virus (A/PR/8) [i.e., Gr-1+ MoDC (i), LN-resident CD8αα+ DC (ii), CD11bhi DC (iii) and CD103+ DC (iv)]. The DC subsets were co-cultured with CFSE-labeled HA533–541 (A/PR/8/34)-specific naïve TCR tg CD8+ T cells (Cl-4) for 4 days. Proliferation profile and total recovery (see inserts) of viable CD8+ T cells stimulated by the MLN-derived DC subsets are depicted. (B) As a control in companion experiments, the DC subsets used in (A) were pulsed with HA210–219 (A/JAPAN/57) and were subsequently co-cultured for 4 days with CFSE-labeled HA 210–219-specific naïve TCR tg CD8+ T cells (Demi-4). These CD8+ TCR tg T cells do not cross-react with the A/PR/8/34 hemagglutinin. (7.09 MB TIF) [file pone.0004204.s012.tif]
